# Case Report: Two New Cases of Chromosome 12q14 Deletions and Review of the Literature

**DOI:** 10.3389/fgene.2021.716874

**Published:** 2021-09-01

**Authors:** Ruizhi Deng, Melysia T. McCalman, Thomas P. Bossuyt, Tahsin Stefan Barakat

**Affiliations:** Department of Clinical Genetics, Erasmus MC University Medical Center, Rotterdam, Netherlands

**Keywords:** chromosome, deletion 12q, clinical genetics, reverse phenotyping, BAFopathies, SMARCC2

## Abstract

Interstitial deletions on the long arm of chromosome 12 (12q deletions) are rare, and are associated with intellectual disability, developmental delay, failure to thrive and congenital anomalies. The precise genotype-phenotype correlations of different deletions has not been completely resolved. Ascertaining individuals with overlapping deletions and complex phenotypes may help to identify causative genes and improve understanding of 12q deletion syndromes. We here describe two individuals with non-overlapping 12q14 deletions encountered at our clinical genetics outpatient clinic and perform a review of all previously published interstitial 12q deletions to further delineate genotype-phenotype correlations. Both individuals presented with a neurodevelopmental disorder with various degrees of intellectual disability, failure to thrive and dysmorphic features. Previously, larger deletions overlapping large parts of the deletions encountered in both individuals have been described. Whereas, individual 1 seems to fit into the previously described phenotypic spectrum of the 12q14 microdeletion syndrome, individual 2 displays more severe neurological symptoms, which are likely caused by haploinsufficiency of the BAF complex member *SMARCC2*, which is included in the deletion. We furthermore perform a review of all previously published interstitial 12q deletions which we found to cluster amongst 5 regions on chromosome 12, to further delineate genotype-phenotype correlations, and we discuss likely disease relevant genes for each of these deletion clusters. Together, this expands knowledge on deletions on chromosome 12q which might facilitate patient counseling. Also, it illustrates that re-analysis of previously described microdeletions syndromes in the next generation sequencing era can be useful to delineate genotype-phenotype correlations and identify disease relevant genes in individuals with neurodevelopmental disorders.

## Introduction

Interstitial deletions of chromosome 12q are rare, often *de novo* occuring, chromosome abnormalities. Various studies described phenotypes of different 12q deletions, including developmental delay, intellectual disability, growth retardation and dysmorphic features (Rauen et al., 2002; Miyake et al., 2004; James et al., 2005; Menten et al., 2007; Niyazov et al., 2007; Adam et al., 2010; Lynch et al., 2011; Vergult et al., 2011; Al-Maawali et al., 2014; Carlsen et al., 2015; De Crescenzo et al., 2015; Cano et al., 2016; Labonne et al., 2016; Alesi et al., 2019; Uehara et al., 2019), but an in-depth overview of all published cases to determine genotype-phenotype correlations and to identify candidate disease-causing genes for most of these microdeletion syndromes is lacking.

We here describe two new cases encountered in our clinic presenting with different, non-overlapping interstitial deletions of chromosome 12q14. The combined deleted region of both cases largely overlaps with previously reported larger deletions (Buysse et al., 2009; Lynch et al., 2011; Nso-Roca et al., 2014; Mc Cormack et al., 2015). The major phenotypical differences identified in both affected individuals triggered us to perform a review of all previously published cases of chromosome 12q14 deletions and 12q deletion in general, with the goal to delineate a phenotype-genotype correlation that would allow improved prediction of clinical phenotypes.

A total of 69 individuals, with various overlapping 12q deletions, were identified in literature, with deletions located across five clusters, with one or several small region of overlap (SROs). Of these, 27 individuals were found with deletions in 12q14. We discuss their phenotypes and the likely disease causing genes of these deletions in the context of the deletions encountered in the two individuals that we describe.

## Methods

### Patient Recruitment

Patients were recruited during their routine visits of the outpatient clinic of the Clinical Genetics Department of the Erasmus MC, Rotterdam, The Netherlands. Written informed consent was obtained from the legal guardians for publication of anonymized medical data and clinical photographs.

### Review Strategy

We searched Pubmed (last assessed: May 2021) for papers describing patients with chromosome 12q deletions, focusing on reports in English and available to the library of the Erasmus MC, and extended our search to papers mentioned in the reference lists of identified papers. We excluded papers that only focused on somatic deletions (e.g., in tumor cells) or papers reporting complex rearrangements and translocations. Identified patients from literature were grouped according to the cytogenetic location of their deletions, and reported symptoms were collected and counted for each group. All genetic coordinates are given in genome build hg19.

### Gene Expression and Probability of Loss-Of-Function Intolerance Analysis

Publically available RNA-seq data from different brain regions and other fetal tissues were collected from the ENCODE project (Consortium, 2012), accession numbers and details are given in Supplementary Table 8. Gene expression levels were normalized based on fragments per kilobase of transcript per million mapped reads (FPKM). pLI scores were downloaded from the gnomAD website (https://gnomad.broadinstitute.org/downloads). Gene expression levels and pLI score were plotted using R packages.

## Results

### Individual 1

**Figure 1:**
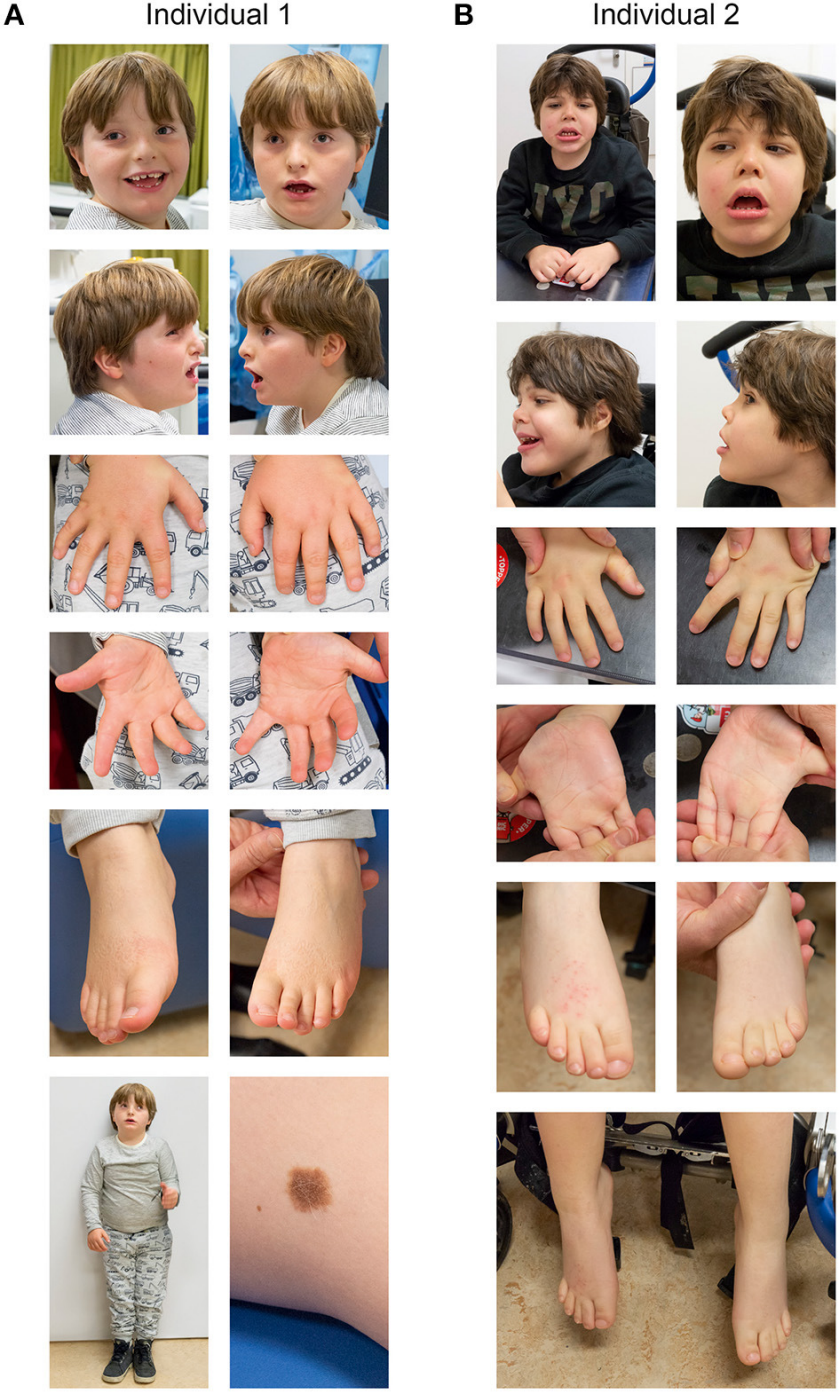
Clinical photographs of individual 1 **(A)** and individual 2 **(B)**. See text for details.

Individual 1 is a 7-year-old male, presenting with developmental delay, moderate intellectual disability and growth retardation (Figure 1). He was the third child of non-consanguineous Dutch parents, born at 36 weeks of gestation, with a birth weight of 1,945 gram (<p3), head circumference of 31.5 cm (p42) and a good start (APGAR 9 and 9 at 1 and 5 min, respectively). Family history was negative for developmental delay or congenital anomalies. Pregnancy was conceived through *in vitro* fertilization (IVF) (his older brother was the product of a fertilized egg conceived during the same IVF cycle and is healthy) and was complicated with recurrent vaginal bleeding and placental insufficiency, with IUGR noticed at 20 weeks of gestation. Screening for TORCHES and routine karyotyping were normal. Frequent airway infections complicated the first year but improved over time, after which psychomotor delay, failure to thrive and dysmorphic features were noticed. He started independent walking at the age of 3 years, with a pronounced exorotation of the legs, requiring orthopedic shoes. First words started around 4 years of age, and at the last investigation at 7 years of age, speech development is severely delayed although progressive with only a few understandable words, requiring special education. His language perception and non-verbal skills are more developed. His tested IQ was 50 (SON-IQ). He reacts very sensitive to sounds, and cannot tolerate crowded places or sudden changes of regularities in his daily activities, indicative of an autism spectrum disorder. Other behavioral issues include occasional temper tantrums when unable to express himself, hyperphagia and incontinence. Physical examination at age of 7 years showed a short stature [110 cm (−3.44 SD)], with normal weight [31 kg (+1.6 SD)] and head circumference of 52.5 cm (+0.15 SD). Outer and inner canthal distance were increased [OCD: 10 cm (>p97); ICD: 3.5 cm (>p97)] with interpupillary distance in the normal range [IPD: 5.5 cm (>p50)]. Neurological examination was normal except a low muscular tone and clumpsy gait. Other findings included upslanting palpebral fissures, a broad nasal bridge, with midfacial hypoplasia, an open mouth posture, with small irregular implanted teeth, a single café-aut-lait spot on the back and a large naevus on the left upper leg which was present since birth (Figure 1A). Previous ultrasound studies of heart and kidneys were normal. Imaging studies of the hips showed coxae valgea antetorta, without signs of osteopoikilosis. A SNP-array identified a *de novo* 4.4 Mb deletion on chromosome 12q14.2-q15 [arr 12q14.2q15 (64,899,031–69,328,844)1×]. This deletion includes 24 protein coding genes including the genes *LEMD3* and *HMGA2*, and overlaps with a region previously linked to a chromosome 12q14 deletion syndrome (Menten et al., 2007).

### Individual 2

Individual 2 is a 7-year-old male with pronounced psychomotor retardation, severe intellectual disability and failure to thrive (Figure 1). He was born after an uneventful pregnancy as the second child to non-consanguineous Dutch parents at 35 + 6 weeks of gestation, with a birth weight of 2,380 gram (p10) and an uncomplicated start of life directly after birth. Family history was negative for developmental delay or congenital anomalies. Feeding difficulties required hospital admission for the first week of life. At 3 months of age developmental delay was first noticed, and at the age of 9 month hypotonia and dysmorphic features were observed. Recurrent airway infections required continuous cotrimoxazol treatment. Independent sitting skills were acquired at 3 years of age, but ambulation, speech, or toilet training never developed. A number of surgical interventions were required due to recurrent hip and knee luxation and flexion contractures. Currently he is wheel chair bound, has no signs of regression and requires PEG feeding. Communication occurs via a language computer aid and pictograms. There is a social smiling with occasional temper tantrums, and more recently involuntary movements. Physical examination at the age of 7 years showed a length of 118 cm (−2.27 SD), weight of 23 kg (−0.99 SD) and head circumference of 52 cm (−0.21 SD); Inner and outer canthal distance were 3 cm (<p75) and 9.5 cm (>p97), respectively. Neurological examination showed pyramidal and extrapyramidal signs, including involuntary movements of the head, trunk and upper extremities. He makes sounds and seems to understand simple instructions to a certain degree. Other findings included a coarse facial appearance with upslanting palpebral fissures, epicanthal folds, a broad nasal bridge, upturned nose, an open mouth posture with widely spaced teeth in the lower jaw and high-arched palate, bilateral simian crease, bilateral 2–3 toe syndactyly and retractile testis. An initial ventricular septum defect closed spontaneously. Brain MRI imaging showed widened central and peripheral liquor spaces. Cerebrospinal fluid analysis and EEG were normal. SNP-array identified a *de novo* 7.3 Mb deletion of chromosome 12q13.2q14.2 [arr 12q13.2q14.2 (56,554,154–63,870,277)×1]. This deletion includes 73 genes, including *SMARCC2*, and is partially overlapping with previously described deletions in 12q13.3q14.2 (Buysse et al., 2009; Lynch et al., 2011; Nso-Roca et al., 2014; Mc Cormack et al., 2015).

## Literature Review of Chromosome 12q Deletions

**Figure 2:**
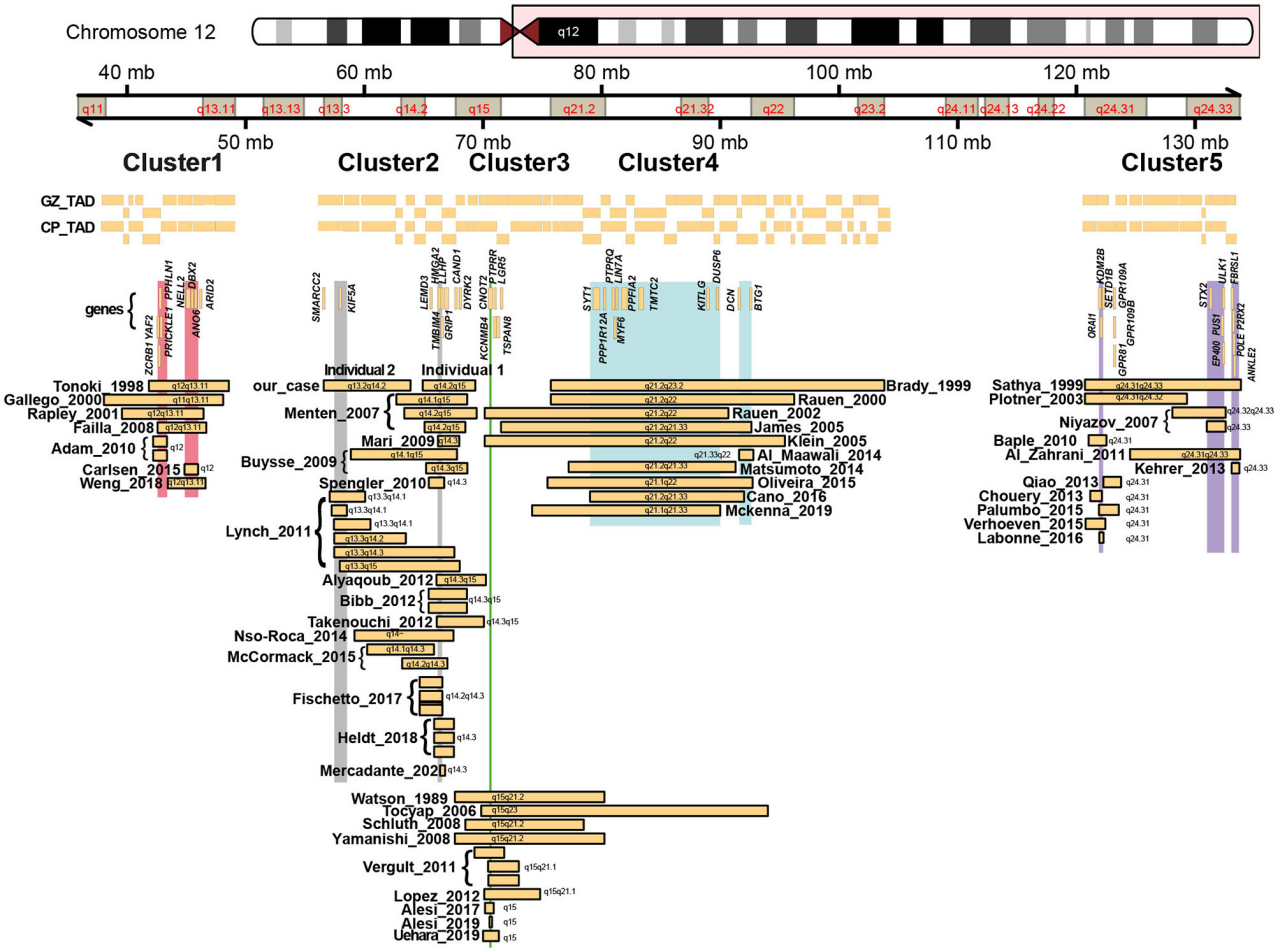
Schematic overview of all previously reported deletions along chromosome 12q, with chromosome ideograms and bands indicated. Horizontal bars represent identified deletions that cluster amongst 5 regions, with names and years referring to the first author and year of publication from the studies in which these deletions were described. Also indicated are topological associated domains (Won et al., 2016) identified from chromatin conformation capture analysis of the germinal zone (GZ) and cortical plate (CP) from human fetal brain. Vertical bars represent small regions of overlaps shared between the different deletions. Genes discussed in the main text or in the Supplemental Materials are indicated.

Both patients differ in their clinical phenotype. Whereas, individual 1 is more mildly affected and is able to walk and has a limited ability to speak, individual 2 is more severely affected, with lack of independent ambulation and speech. Both individuals have a short stature, failure to thrive and a few shared dysmorphic features (Figure 1), but clear differences in their phenotype manifest. As both deletions encountered in these individuals were non-overlapping but having breakpoints in proximity (e.g., within ~1 Mb), and previously a number of deletions were described that encompass large parts of both deletions (Buysse et al., 2009; Lynch et al., 2011; Nso-Roca et al., 2014; Mc Cormack et al., 2015), we set out to review the literature to identify likely genes involved in the phenotypic differences and to further delineate the genotype-phenotype correlation associated with chromosome 12q deletions in general. We found 69 published individuals with 12q deletions and their clinical phenotypes (Supplementary Table 1). Overlapping deletions clustered in 5 regions at 12q11q13.1, 12q13q15, 12q13.3q23.1, 12q21.1q23.2, and 12q22q24.33 (Figure 2). We here focus on the cluster at 12q13q15, which includes the two deletions encountered in individual 1 and 2, and discuss the other clusters in more detail in the Supplemental Material and Supplementary Tables 4–7.

### Individual 1 Fits the Spectrum of 12q13q15 Deletions Encompassing the 12q14 Microdeletion Syndrome

**Figure 3:**
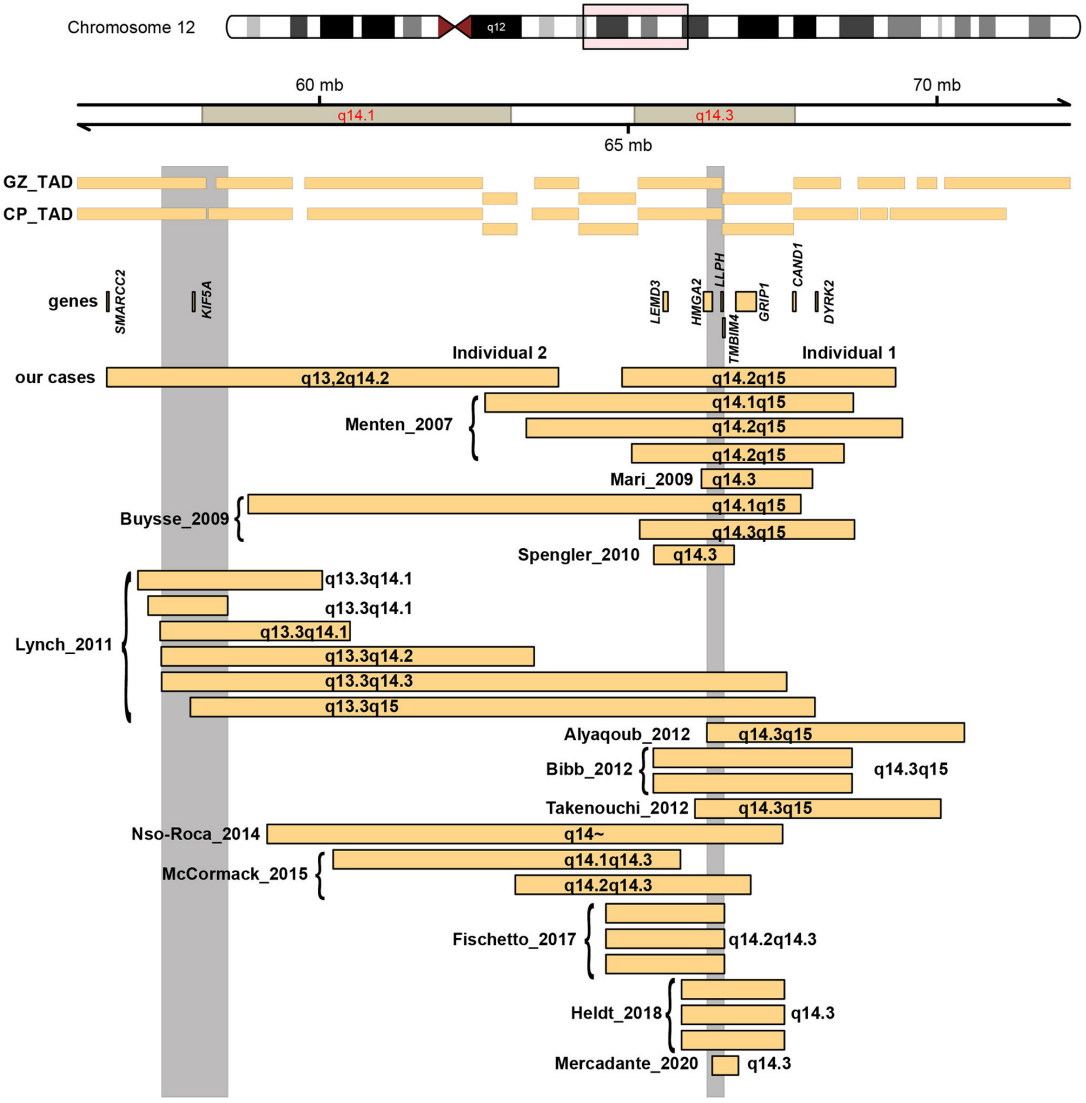
As Figure 2, but now zoomed-in at cluster 2 at 12q14.

Twenty-seven individuals (14 females, and 13 males) with overlapping deletions located within 12q13q15 have been previously reported with varying degrees of global developmental delay/intellectual disability, growth retardation and short stature as the main phenotype (Menten et al., 2007; Buysse et al., 2009; Mari et al., 2009; Spengler et al., 2010; Lynch et al., 2011; Alyaqoub et al., 2012; Bibb et al., 2012; Takenouchi et al., 2012; Fischetto et al., 2017; Heldt et al., 2018; Mercadante et al., 2020; Figure 3, Supplementary Table 2).

The deletion encountered in individual 1 overlaps with 22 reported deletions which have been associated with a 12q14 microdeletion syndrome (Menten et al., 2007; Buysse et al., 2009; Mari et al., 2009; Spengler et al., 2010; Lynch et al., 2011; Alyaqoub et al., 2012; Bibb et al., 2012; Takenouchi et al., 2012; Nso-Roca et al., 2014; Mc Cormack et al., 2015; Fischetto et al., 2017; Heldt et al., 2018; Mercadante et al., 2020), and the boundaries largely overlap with seven of these previously published deletions (Menten et al., 2007; Buysse et al., 2009; Bibb et al., 2012; Takenouchi et al., 2012), with an SRO located in 12q14.3. Menten et al. described three individuals, with a core phenotype of mild intellectual disability, low birth weight with failure to thrive in early infancy, proportionate short stature and osteopoikilosis (the latter is in combination with multiple subcutaneous nevi or nodules also known as Buschke-Ollendorf syndrome) (Hellemans et al., 2004). Dysmorphic features between the three individuals were not consistent, and included synophrys, mild hypertelorism, broad and high nasal bridge, micrognathia, maxillary overbite, round face with deep-set eyes, bushy eyebrows, thin lips, and a triangular face with widely spaced eyes. In a follow-up study, two additional individuals with overlapping deletions were described (Buysse et al., 2009), that presented with a similar phenotype. Interestingly, in that report, also an intragenic deletion of *HMGA2* was found in a boy with proportionate short stature, and this deletion segregated with this phenotype in this family, indicating that *HMGA2* is responsible for the short stature seen in individuals with 12q14 deletions. His neurodevelopment was normal (Buysse et al., 2009). Bibb et al. reported a mother and a daughter sharing a deletion in this region (Bibb et al., 2012). The daughter presented with failure to thrive, hypertelorism, upturned nose, mild micronathia, and clinodactyly, and was suspected of Silver-Russel syndrome. She also had physical and language delays, mild intellectual disability, behavioral problems, and sleep disturbance. The mother also presented early in life with failure to thrive, a short stature, mild language delay, microcephaly, a café aut lait spot, mild clinodactyly, upslanting palpebral fissures, and osteopoikilosis. The individual described by Takenouchi et al. showed failure to thrive, short stature, no relative macrocephaly, and surprisingly an appropriate psychomotor development at 29 month of age without autism (Takenouchi et al., 2012). Lynch et al. described 6 additional cases with 12q14 deletions, of whom only two shared the SRO in 12q14.3 (Lynch et al., 2011). Both deletions were larger than the one found in individual 1 in this report. Both individuals had failure to thrive, one individual displayed moderate developmental delay and expressive speech delay, whereas the second individual had severe developmental delay, with autism and absence of speech.

Taken together, the phenotype of moderate developmental delay and failure to thrive of individual 1 seems to fit well to the spectrum previously described for similar range deletions, although osteopoikilosis has not yet been noticed in this individual. The key features of failure to thrive, short stature and osteopoikilosis found in 12q14 deletions seem by current day's knowledge to be explained by haploinsufficiency of *HMGA2* and *LEMD3*, respectively.

*HMGA2*, located at band 12q14.3, encodes an architectural transcription factor, and is a critical component of the enhanceosome. Based on studies performed after the reports on the various 12q14 deletions, *HMGA2* has now been implicated as one of the causes of Silver-Russel syndrome (OMIM #618908), which is characterized by intrauterine growth retardation, postnatal feeding difficulties and growth failure, with dysmorphic features including a relative macrocephaly at birth and a triangular face with prominent forehead. Support for *HMGA2* being implicated in growth originally came from GWAS studies (Weedon et al., 2007) and mouse models (Zhou et al., 1995). In the 12q14 deletions discussed above *HMGA2* was hypothesized to be causal for failure to thrive and short statures (Menten et al., 2007; Buysse et al., 2009; Bibb et al., 2012; Takenouchi et al., 2012). Mari et al. (2009), Heldt et al. (2018), and Mercadante et al. (2020) described individuals with a clinical phenotype reminiscent to Silver-Russel syndrome, with deletions that included *HMGA2* and a limited number of additional genes (Mari et al., 2009; Heldt et al., 2018; Mercadante et al., 2020). Smaller genetic alterations, solely affecting *HMGA2*, including a 7bp deletion that affects splicing (De Crescenzo et al., 2015), a 7.3Kb deletion of exon 1 and 2 (Leszinski et al., 2018), other small deletions (Buysse et al., 2009), and missense and truncating variants have subsequently been found in Silver-Russel syndrome like cases (Abi Habib et al., 2018; Hübner et al., 2020), pinpointing *HMGA2* as the causative gene. In agreement with this, all cases with a deletion encompassing this gene have a short stature (Zhou et al., 1995; Weedon et al., 2007; Buysse et al., 2009; Alyaqoub et al., 2012; Supplementary Table 2).

Osteopoikilosis is linked to loss-of-function of *LEMD3* (Hellemans et al., 2004; Mumm et al., 2007; Zhang et al., 2009), a gene encoding a LEM-domain containing protein that functions to antagonize BMP and transforming growth factor-beta signaling (Hellemans et al., 2004). Six of the twenty-seven individuals from literature with deletions in this cluster had osteopoikilosis and in all 6 cases LEMD3 was deleted. However, LEMD3 was deleted in 16 out of the 27 cases, and in the remaining cases with LEMD3 deletion, including the case described herein, no osteopoikilosis was reported at the moment of investigation (Supplementary Table 2). It is unknown whether those cases later on developed this condition.

Interestingly, the neurodevelopmental phenotypes associated with deletions of 12q14 cannot be explained by haploinsufficiency of either *HMGA2* or *LEMD3* alone, as individuals that only have gene specific alterations either showed normal development (Buysse et al., 2009; Leszinski et al., 2018; Hübner et al., 2020), or only mild delay (Mercadante et al., 2020). Psychomotor retardation is also rarely encountered in Silver-Russel syndrome. This indicates that neurodevelopmental delay, observed in most cases of 12q14 deletions, might be caused by other genes in or outside the SRO. The minimal SRO overlapping with the deletion found in individual 1 is 281,741 bp, and next to *HMGA2* contains the genes *LLPH* and *TMBIM4*. Both have not yet been associated with human disease. *LLPH* encodes an intrinsically disordered protein predicted to function in the nucleolus, possibly as a molecular hub for protein-protein interactions (Yu et al., 2016). *TMBIM4* is a highly conserved Golgi membrane protein that inhibits apoptosis and promotes Ca(2+) release from intracellular stores (Saraiva et al., 2013). Both genes however have a low pLI score (Karczewski et al., 2020), making it unlikely that haploinsufficiency of these genes causes a human disorder.

A frequently discussed candidate gene for the neurodevelopmental phenotypes, outside of the SRO is *GRIP1. GRIP1*, encodes a Glutamate-receptor interacting protein 1 that is widely expressed in brain and involved in glutamergic synapse transmission. It has been reported to function in modulating long-term synaptic depression in the cerebellum (Takamiya et al., 2008; Alyaqoub et al., 2012). Bi-allelic variants in *GRIP1* were found to segregate with Fraser syndrome which is characterized by cryptophthalmos, syndactyly, and abnormalities of the respiratory and urogenital tract (Van Haelst et al., 2008; Vogel et al., 2012). *GRIP1* has been suggested as a candidate gene for developmental delay and learning difficulties observed in 12q14 deletions (Menten et al., 2007; Dória et al., 2020). However, as *GRIP1* is not deleted in all individuals (18 out of 27 in this cluster) with neurodevelopmental delay in this cluster (Supplementary Table 2), the individual described by Takenouchi et al. did not display psychomotor retardation despite *GRIP1* deletion and multiple heterozygous loss-of-function variants are found in healthy controls from gnomAD (pLI = 0.50828) (Karczewski et al., 2020), it seems unlikely that *GRIP1* is implicated in neurodevelopmental delay in 12q14 deletions.

The precise genetic cause of neurodevelopmental delay in 12q14 deletion thus remains to be determined. Within the genes covered by the deletion of individual 1, *CAND1* and *DYRK2* have a high pLI score (pLI = 1.0 and pLI = 0.99751, respectively), indicating that they could be susceptible to haploinsufficiency. Both genes are expressed in brain, and are each deleted in 11 and 5 cases, respectively, making them interesting candidate genes to explore further in future studies.

### The More Severe Clinical Phenotype of Individual 2 Is Likely Explained by Haploinsufficiency of SMARCC2

Whereas, individual 1 is well-explained by the deletion and the spectrum of phenotypes associated with the 12q14 microdeletion syndrome, individual 2 is more severely affected than what has been described for individuals with other large deletions in this region (Buysse et al., 2009; Lynch et al., 2011; Nso-Roca et al., 2014; Mc Cormack et al., 2015). As mentioned above, the individuals with the largest deletions described by Lynch et al. (2011) that overlap with the SRO in 12q14, presented with moderate to severe developmental delay, but both individuals achieved independent walking. Individual 4 of Lynch et al. (2011) which harbors a deletion that overlaps large parts of the deleted region in individual 2 from this report, displayed more severe delay and autism, but developed the use of single words and was reported to be extremely active. Also the other three cases of Lynch et al. (2011) were reported to be moderately delayed, with speech delay and absence of speech in one individual, but also acquired independent ambulation. Similar, the largest deletions described by Buysse et al. (2009) is milder affected and able to use many words. The individual described by Nso-Roca had learning problems, besides failure to thrive, and the individual described by Mc Cormack in which the deletion did not contain *HMGA2*, had relative macrocephaly and autism, wild mild delayed gross and fine motor skills and language delay showing encouraging improvements in all aspects of development over time. The 6 cases described by Lynch et al. (2011) possibly point to another SRO at 12q13.3q14.1, containing 34 genes (Supplementary Table 3), which overlaps with the deletion found in individual 2, which covers 73 genes.

**Figure 4:**
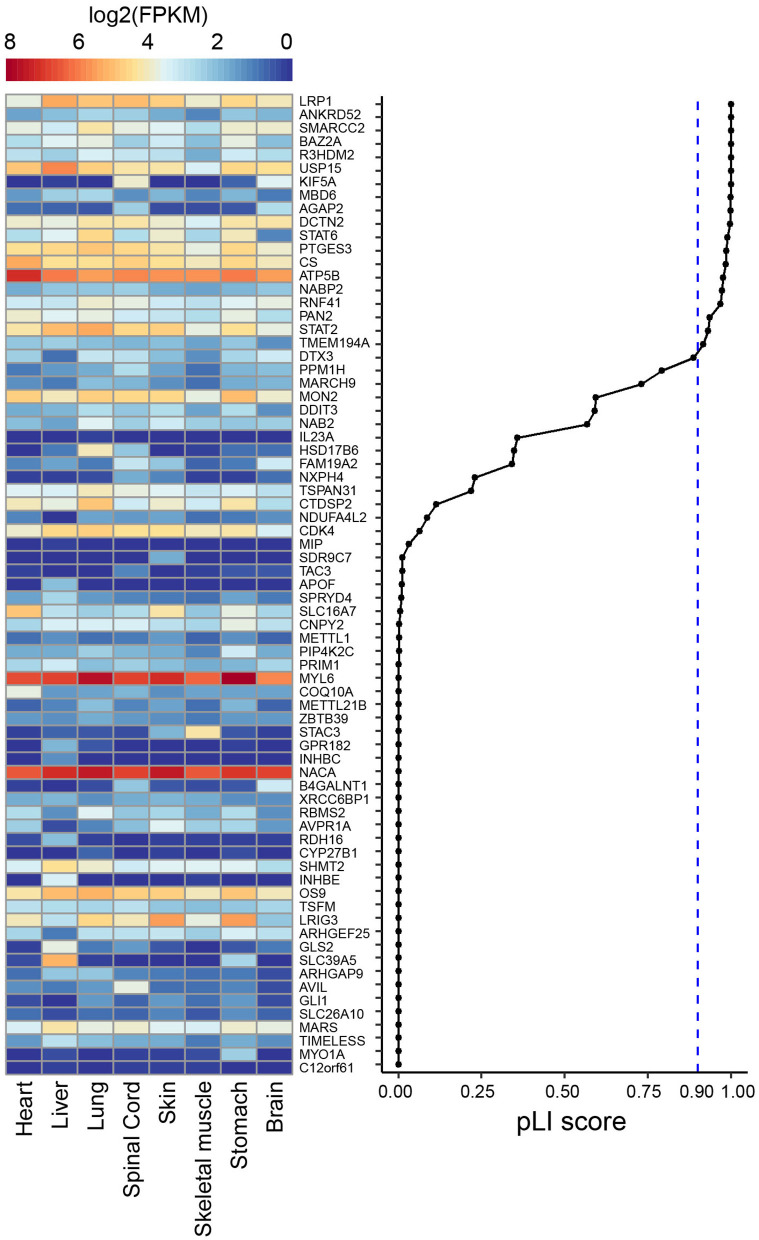
Heat map showing publically available gene expression data (in log_2_ FPKM) amongst 8 human fetal tissues from ENCODE for the 73 genes that are included in the deletion found in individual 2. Genes are ranked according to their pLI score from gnomAD (Karczewski et al., 2020).

To further understand the more severe phenotype of individual 2, we manually assessed all 73 genes included in the deletion searching for known phenotypes, determined their pLI scores and assessed whether there was evidence of expression of these genes in fetal human tissues including brain (Figure 4). We found 19 genes with a pLI > 0.9, indicating that they are intolerant for loss-of-function and could thus be involved in causing a phenotype, when deleted on a single allele. These genes included protein coding genes previously not associated with human disorders or with unclear associations, including *LRP1, R3HDM2, ANKRD52, BAZ2A, R3HDM2, USP15, MBD6, AGAP2, DCTN2*, and the two OMIM genes *KIF5A* and *SMARCC2. KIF5A* (OMIM #604187) is associated with a autosomal dominant form of hereditary spastic paraplegia, with lower limb spasticity, and hyperreflexia, and variable involvement of the upper limbs beginning in childhood or young adulthood. A complicated phenotype with other neurological symptoms can also be observed in individuals with *KIF5A* variants, and genotype-phenotype correlations seem to depend on the *KIF5A* domain in which a variant is found (De Boer et al., 2021). Most of the variants encountered are missense variants, possibly pointing to a dominant-negative mechanism. Although *KIF5A* could possibly explain spasticity in individual 2, the fact that this gene is also deleted in other more mildly affected cases with deletions of the SRO at 12q13.3q14.1, make it less likely that KIF5A could explain the more severe phenotype of individual 2.

In contrast, based on this analysis, *SMARCC2* appeared as a likely disease implicated gene. *SMARCC2* is a member of the BRG1-associated factor (BAF) chromatin-remodeling complex that plays an essential role in the regulation of gene expression and higher-order chromatin organization by modulating the nucleosome and changing chromatin accessibility (Alfert et al., 2019). Aberrations of complex members result in BAFopathies, that present with syndromic neurodevelopmental disorder, including Coffin-Siris syndrome and Nicolaides-Baraitser syndrome. Recently *de novo* variants in *SMARCC2* were found to cause a syndrome with intellectual disability and developmental delay (Machol et al., 2019) (OMIM #618362). Machol et al. (2019 described 15 unrelated individuals with mild to severe intellectual disability, developmental delay with pronounced speech delay with 7 individuals lacking language, behavioral abnormalities, growth retardation in 6 individuals, feeding difficulties in 8 individuals, muscle tone abnormalities including hypotonia and spasticity in 13 individuals, and movement disorders found in two individuals. Behavioral problems included aggression and self-injurious behavior, hyperactivity, hypersensitivity to touch, sleep disturbances, and obsessive and rigid behavior. Dysmorphic features included hypertrichosis, thick eyebrows/prominent supra-orbital ridges, thin upper or thick lower vermillion and upturned nose, suggesting overlap with Coffin-Siris and Nicolaides-Baraitser syndromes. Encountered *SMARCC2* variants included both missense variants and protein truncating alterations, including splice site alterations. To our knowledge, no whole gene deletion of *SMARCC2* has previously been described, but given the overlap in phenotypes (including severe intellectual disability, absence of speech, muscle tone abnormalities, movement disorders and overlap in dysmorphic features) and the loss-of-function mechanism for some of the variants reported by Machol et al., we propose that *SMARCC2* is the most likely disease causing gene in individual 2. Additional genes contained in the deletion and with impact on the phenotype which have not been identified yet cannot be ruled out.

## Discussion

We report two individuals with previously undescribed interstitial deletions at 12q14. In their diverse phenotype they triggered a review of all previously described chromosome 12q deletion. Whereas, we find that the phenotype of individual 1 is compatible to what has previously been described for the 12q14 microdeletion syndrome (Menten et al., 2007), individual 2 is more severely affected. This difference in phenotype seems to be caused by haploinsufficiency of the BAF chromatin remodeling complex member *SMARCC2*, which has recently been implicated in a neurodevelopmental disorder with overlaps to Coffin-Siris syndrome (Machol et al., 2019) (OMIM #618362). To our knowledge, individual 2 is the first case of a whole gene deletion of *SMARCC2*, thereby expanding the disease causing molecular spectrum of this recently identified syndrome. As other BAFopathies are characterized by complex specific DNA-methylation signatures (Aref-Eshghi et al., 2018), it will be interesting to investigate whether such an epi-signature can also be determined for pathogenic *SMARCC2* variants, and whether an identical signature would be obtained in individual 2, which would further support the diagnosis. The case of individual 2, and also the review of the other deletion clusters amongst chromosome 12q, illustrate that a regular re-interpretation of previously diagnosed microdeletions can be useful to gain new insights in the mechanisms leading to disease phenotypes in the affected individuals. In the case of individual 2, the initial diagnosis of a 12q13.2q14.2 deletion was made years before *SMARCC2* was identified as a disease gene. Similarly, re-interpretation and additional fine mapping of previously suggested microdeletion syndromes at 12q15 and 12q24.31 has now led to the conclusions that most of the phenotypes associated with these microdeletion syndromes are caused by alterations of the genes *CNOT2* (Alesi et al., 2017) and *SETD1B* (Weerts et al., 2021), respectively. It is expected that further progress in genetics, cell biology and personalized medicine will advance options to influence the phenotypes by gene-specific or pathway driven therapeutics. It is thus remains crucial to identify disease causing genes in previously diagnosed microdeletion cases. We suggest that a long term follow-up of patients with microdeletions can be helpful for this and can also improve the genetic counseling in these cases.

## Data Availability Statement

Publicly available datasets were analyzed in this study. This data can be found at: ENCODE project (see Supplementary Table 8).

## Ethics Statement

Ethical review and approval was not required for the study on human participants in accordance with the local legislation and institutional requirements. Written informed consent to participate in this study was provided by the participants' legal guardian/next of kin. Written informed consent was obtained from the minor(s)' legal guardian/next of kin for the publication of any potentially identifiable images or data included in this article.

## Author Contributions

MM performed the initial literature review. RD performed bioinformatics analysis, literature review and wrote parts of the manuscript, and was supported by TPB. TSB conceived the study, performed clinical investigations, and wrote the manuscript together with RD. All authors contributed to the article and approved the submitted version.

## Conflict of Interest

The authors declare that the research was conducted in the absence of any commercial or financial relationships that could be construed as a potential conflict of interest.

## Publisher's Note

All claims expressed in this article are solely those of the authors and do not necessarily represent those of their affiliated organizations, or those of the publisher, the editors and the reviewers. Any product that may be evaluated in this article, or claim that may be made by its manufacturer, is not guaranteed or endorsed by the publisher.

## Supplementary Material

Supplementary Figure 1 As Figure 2, but now zoomed-in at cluster 1.

Supplementary Figure 2 As Figure 2, but now zoomed-in at cluster 3.

Supplementary Figure 3 As Figure 2, but now zoomed-in at cluster 4.

Supplementary Figure 4 As Figure 2, but now zoomed-in at cluster 5.

Supplementary Table 1 Summary of reported 12q deletions.

Supplementary Table 2 Summary of overlapping clinical features of 27 reported individuals with deletions at 12q13q15 (12q14). a, G-banding; b, BAC; c, array-CGH; d, SNP-array.

Supplementary Table 3 Genes included in SROs.

Supplementary Table 4 Summary of clinical features of 8 reported individuals with deletions at 12q11.1q13.1. a, G-banding; b, BAC; c, array-CGH; d, SNP-array.

Supplementary Table 5 Summary of overlapping clinical features of 11 reported probands with deletions at 12q13.3q23.1. a, G-banding; b, BAC; c, array-CGH; d, SNP-array; e, Microsatellite Marker Analysis.

Supplementary Table 6 Summary of overlapping clinical features of 10 reported individuals with deletions at 12q21.1q23.2. a, G-banding; b, BAC; c, array-CGH; d, SNP-array.

Supplementary Table 7.1 Summary of overlapping clinical features of 13 reported individuals with deletions at 12q22q24.33. a, G-banding; b, BAC; c, array-CGH; d, SNP-array.

Supplementary Table 7.2 Summary of overlapping clinical features of 7 reported individuals with deletions at 12q24.31.

Supplementary Table 7.3 Summary of overlapping clinical features of 7 reported individuals with deletions at 12q24.31.

Supplementary Table 8 Public data used.
