# Supplementary figures and images for: Case Report: Two New Cases of Chromosome 12q14 Deletions and Review of the Literature
Source: Front Genet. 2021 Sep 1;12:716874. doi: 10.3389/fgene.2021.716874 (PMC8441011; doi:10.3389/fgene.2021.716874)

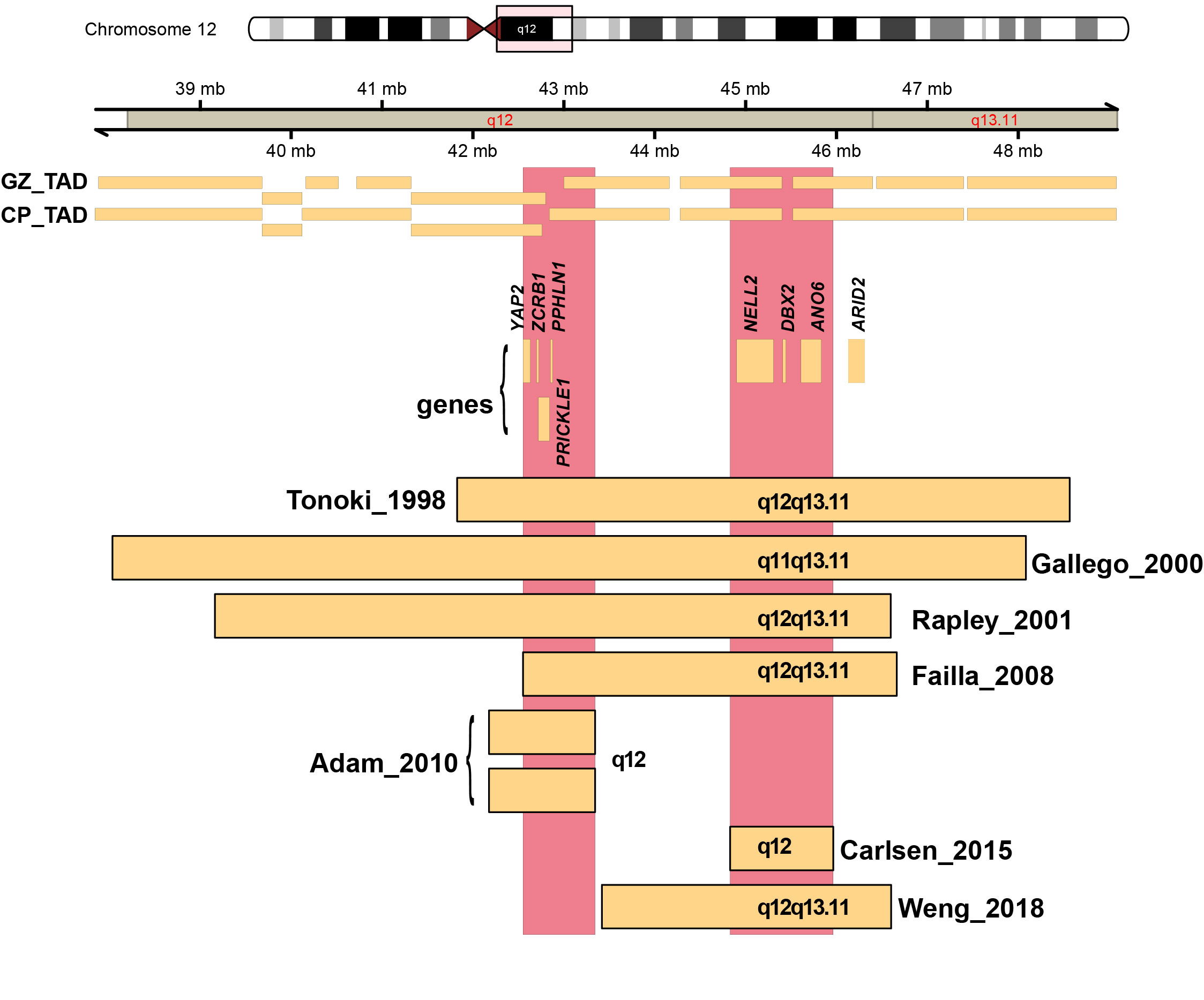

Supplement: Supplementary Figure 1 — As Figure 2, but now zoomed-in at cluster 1. [file Image_1.JPEG]

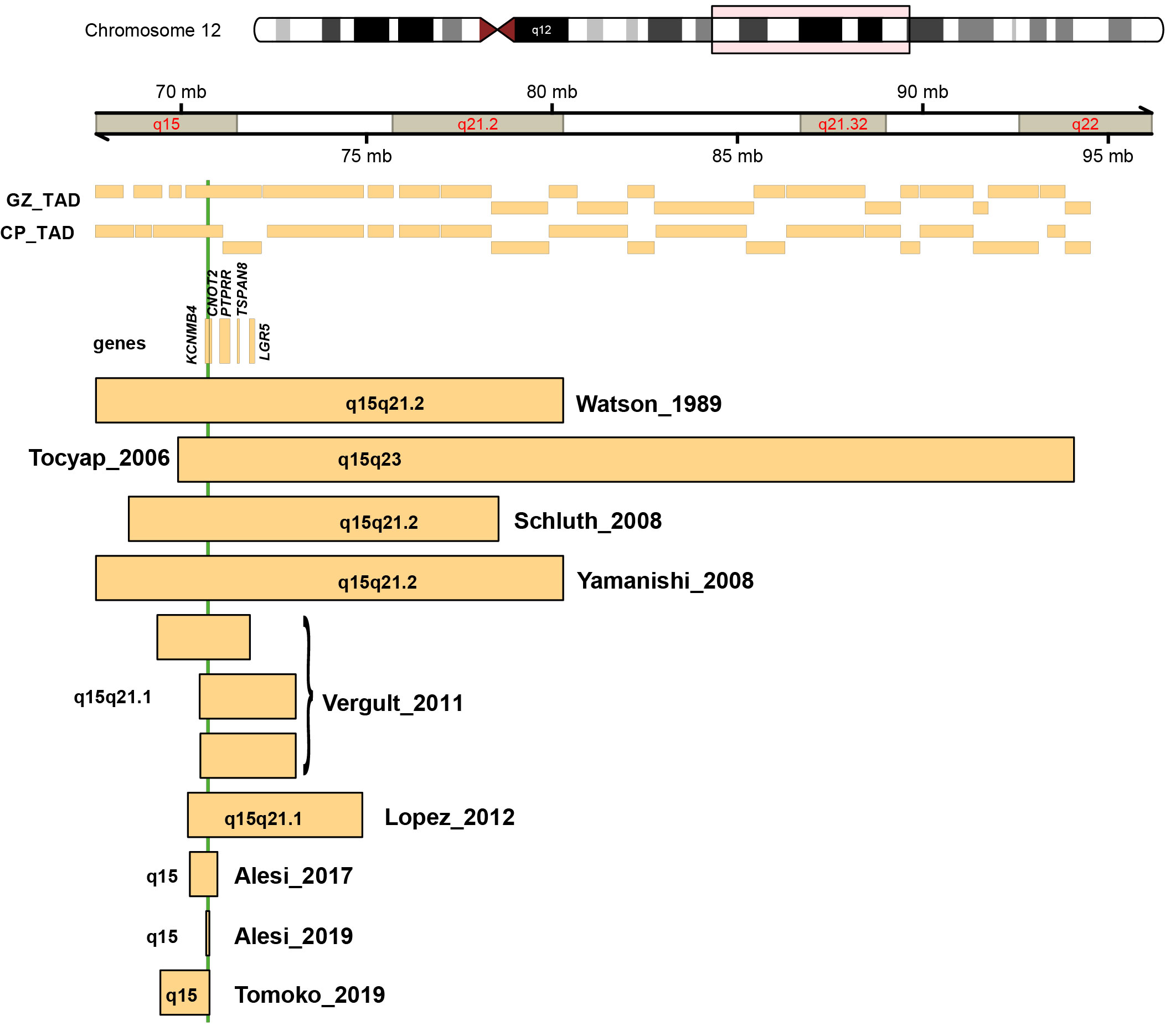

Supplement: Supplementary Figure 2 — As Figure 2, but now zoomed-in at cluster 3. [file Image_2.JPEG]

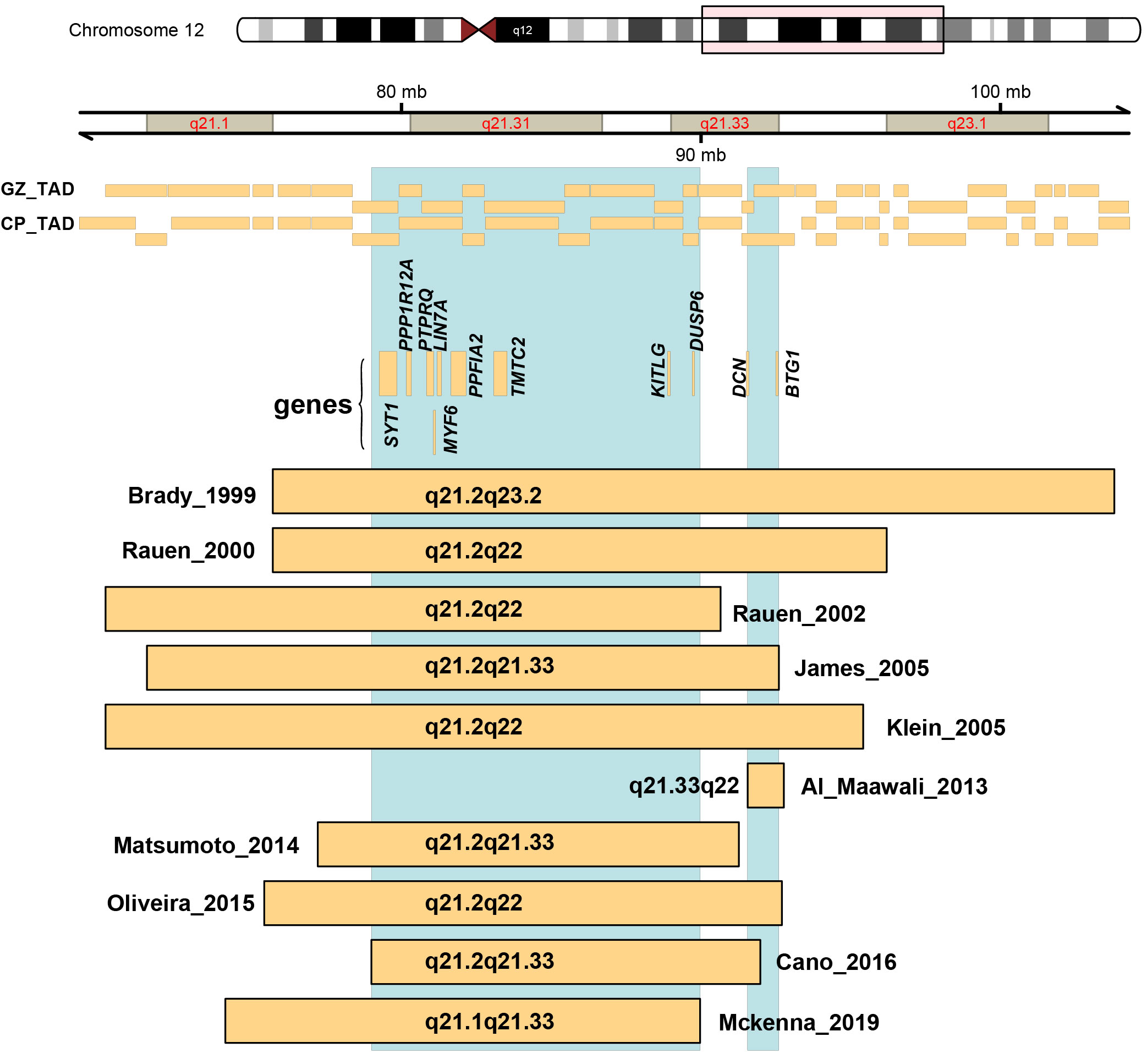

Supplement: Supplementary Figure 3 — As Figure 2, but now zoomed-in at cluster 4. [file Image_3.JPEG]

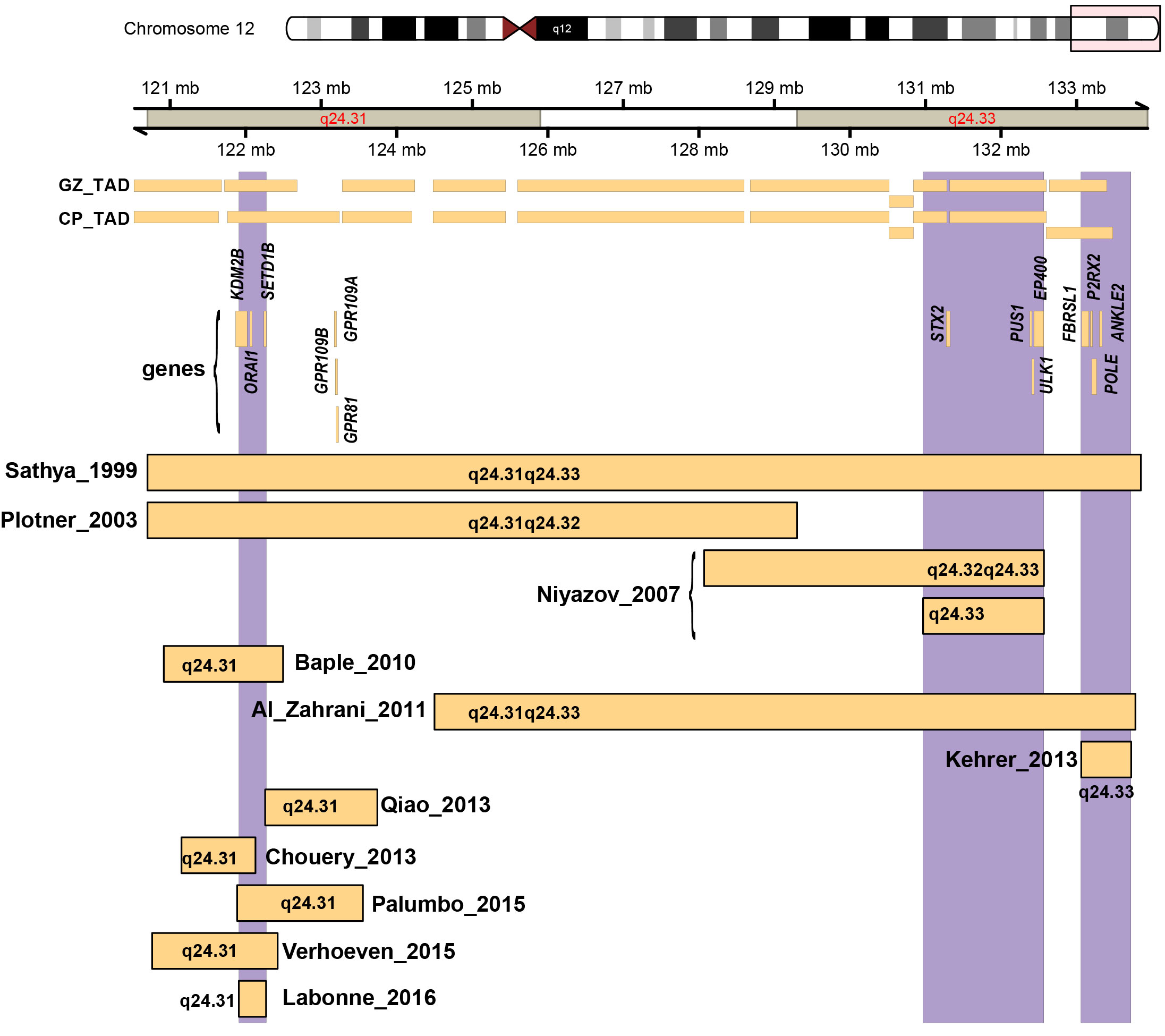

Supplement: Supplementary Figure 4 — As Figure 2, but now zoomed-in at cluster 5. [file Image_4.JPEG]
