# Supplementary material for: Case Report: Two New Cases of Chromosome 12q14 Deletions and Review of the Literature
Source: Front Genet. 2021 Sep 1;12:716874. doi: 10.3389/fgene.2021.716874 (PMC8441011; doi:10.3389/fgene.2021.716874)
Supplement: Supplementary file 6 [file Data_Sheet_2.docx]

**Supplementary Material:**

*Cluster 1- 12q11q13.12*

Eight individuals (7 males, and 1 female) with partially overlapping deletions spanning 12q11.1q13.1 have been reported presenting with global developmental delay/intellectual disability, hypotonia and mild to severe growth and psychomotor retardation (Rapley et al., 2001;Miyake et al., 2004;Failla et al., 2008;Adam et al., 2010;Carlsen et al., 2015;Weng et al., 2018) (**Supplementary Figure 1*,* Supplementary Table 4**). All but one deletion occurred *de novo,* with healthy, non-consanguineous parents. In one case, the deletion was inherited from a mildly affected father (Adam et al., 2010) (**Supplementary Figure 1*,* Supplementary Table 4**). In two cases, intrauterine growth retardation (IUGR) was observed (Tonoki et al., 1998;Failla et al., 2008). All affected individuals showed various dysmorphic features, including a broad/prominent forehead, slanted palpebral fissures, abnormally shaped nose, low set ears and hand/finger deformities (**Supplementary Table 4**). Deformities of hand and feet included small hands, proximal inserted thumbs, a single palmar crease, fifth finger clinodactyly and 2^nd^/3^rd^ syndactyly. Eye and testes anomalies, hypotonia and delayed speech were also present in at least 3 individuals. Feeding difficulties were also often described.

6 individuals (including the index and his father described by Adam et al. (Adam et al., 2010)) share an SRO of 0.79 Mb, harboring the 4 genes *YAF2*, *ZCRB1, PPHLN1,* and *PRICKLE1*, which were proposed to be implicated in a 12q12 deletion syndrome (Adam et al., 2010) (**Supplementary Figure 1, Supplementary Table 3**). In contrast, a 1.13 Mb *de novo* deletion reported by Carlsen et al. (Carlsen et al., 2015) in an individual displaying the same phenotypic spectrum, contains the protein coding genes *NELL2, DBX2* and *ANO6*. This deletion is located more telomeric than the smallest 1.17 Mb deletion reported by Adam et al., but is also overlapping with other described deletions in the 12q12 region. Another 3.18 Mb deletion in a patient with a similar phenotype described by Weng et al. overlaps this 1.13 Mb deletion and shares a large overlap with other 12q12 deletions, but also does not harbor the minimal region determined by Adam et al. Therefore, it cannot be excluded that also this smaller 1.13 Mb region and its surroundings contain genes relevant for the phenotypes of the other 12q12 deletion individuals.

The proximal 0.79 Mb SRO contains *YAF2*, *ZCRB1, PPHLN1,* and *PRICKLE1. YAF2* encodes a zinc finger containing protein that functions in the regulation of transcription and is implicated in early embryonic development and organogenesis for its function in the regulation of apoptosis (Stanton et al., 2006). Miyake et al. Failla et al. and Adam et al. proposed that this gene may be involved in growth retardation observed in patients with 12q12 deletions. Adam et al. further suggested that the extent of the growth retardation is accentuated by the deletion of other genes in the region which may account for the observed variation. Of interest is that YAF2 interacts with YY1 (Basu et al., 2014), a multifunctional transcription factor which is implicated in an intellectual disability syndrome (Gabriele et al., 2017) and plays an important role in gene regulation (Verheul et al., 2020).

*ZCRB1* encodes a multifunctional nuclear factor that has a RNA recognition motif. The protein may act as a member of splicesomes and has been proposed to be a hepatocarcinoma candidate oncogene (Wang et al., 2007;Adam et al., 2010). The case described by Rapley et al. was diagnosed with a Wilms tumor. It is unknown whether *ZCRB1* haploinsufficiency contributed to tumor development in this individual, but as malignancies were not reported in the other cases, this seems unlikely.

*PPHLN1* encodes a structural protein involved in epithelial differentiation which becomes sequentially incorporated into the cornified cell envelope during terminal differentiation of keratinocyte at the outer layers of the epidermis (Kazerounian and Aho, 2003). A syringocystademona papilliferum has been described in the case presented by Failla et al., but given that dermatologic abnormalities have not been found in other individuals with 12q12 deletions, it remains unknown whether haploinsufficiency of *PPHLN1* has a significant phenotypic effect (Adam et al., 2010).

*PRICKLE1,* a nuclear receptor, belongs to a family of genes that are core constituents of the planar cell polarity signaling pathway, which is key to establish cell polarity during embryonic development. PRICKLE proteins are expressed in brain and are known to bind to and functionally interact with REST, an essential regulator of neural genes (Shimojo and Hersh, 2003;2006). Bi-allelic variants in *PRICKLE1* have been associated with epileptic encephalopathy, whereas heterozygous variants have been reported in neural tubes defects, corpus callosum agenesis, polymicrogyria, autism spectrum disorders and structural birth defects (Bassuk and Sherr, 2015;Gibbs et al., 2016;Mastrangelo et al., 2018;Todd and Bassuk, 2018). Therefore, haploinsufficiency of *PRICKLE1* might contribute to intellectual disability in some of the 12q12 individuals (Failla et al., 2008;Adam et al., 2010).

The distal SRO contains the protein coding genes *NELL2, DBX2,* and *ANO6*, of which some seem likely to also contribute to the phenotype of individuals harboring deletions of both SROs.

*ANO6* encodes Anoctamin 6, of which bi-allelic loss-of-function mutation cause Scott syndrome, a rare inherited bleeding disorder (Millington-Burgess and Harper, 2019). None of the individuals were reported to have bleeding abnormalities, and thus the involvement of this gene in the phenotype seems unlikely.

*NELL2* encodes the neural epidermal growth factor-like like 2, which binds protein kinase C. It is widely expressed in brain, plays a role in axon guidance, neuronal migration (Jaworski et al., 2015;Pak et al., 2020) and various other brain related processes (Kim et al., 2002;Aihara et al., 2003).

*DBX2* encodes developing brain homeobox protein 2, which is a highly conserved protein involved in primary neurogenesis. In both flies and mice, loss of *Dbx2* function resulted in a movement phenotype (Lanuza et al., 2004;Lacin et al., 2009). Haploinsufficiency of these gene have so far not been described in monogenic disease, but given their involvement brain related processes, *NELL2* and *DBX2* seem plausible candidates at least contributing to the neurodevelopmental phenotype encountered in patients with 12q12 deletions.

Interestingly, ~165 kb telomeric to the distal breakpoint of the 1.13 Mb deletion described by Carlsen et al. (Carlsen et al., 2015) which defines the distal boundary of this SRO, *ARID2* is located. *ARID2* is an intrinsic component of the polybromo-associated BAF (PBAF) complex, a subcomplex of the ATP-dependent SWI/SNF chromatin remodeling complex. In 2015 *de novo* variants in *ARID2* were for the first time associated with Coffin-Siris syndrome (Shang et al., 2015), which presents with short stature, sparse hair, mild to severe intellectual disability, coarse facial features, variable behavioral anomalies, fifth digit clinodactyly with small nails and several dysmorphic facial features including micrognathia or retrognathia, low set or posteriorly rotated ears, epicanthal folds, down slanting palpebral fissures, highly arched palate, and frontal bossing. More cases have subsequently been described (Bramswig et al., 2017;Van Paemel et al., 2017), and all are reminiscent to the case described by Carlsen et al. (Carlsen et al., 2015), which showed global developmental delay, failure to thrive, behavioral issues and various dysmorphic features, including a broad forehead, strabismus, downslanting palpebral fissures, hypertelorism, small and lowset ears, depressed nasal bridge with a smooth philtrum, wide mouth and fifth finger clinodactyly. As the deletion described by Carlsen et al. (Carlsen et al., 2015) disrupts the boundary of two topologically associated domains flanking the *ARID2* locus, which were identified by chromatin conformation capture studies on the germinal zone of human fetal brain (Won et al., 2016) (**Supplementary Figure 1**), it is tempting to speculate that this deletion might affect gene regulation of *ARID2*, thereby causing a Coffin-Siris-like phenotype. This could argue that deletion of *NELL2* and *DBX2* itself might not be causal, but it will require further functional investigation to test this hypothesis. Nonetheless, as *ARID2* is included in five of the eight deletions in this cluster (with the only exception being the case and his mildly affected father described by Adam et al. 2010 and the case of Carlsen et al. 2015), and the association between *ARID2* and Coffin-Siris syndrome was published later than most of the deletions, it is likely that heterozygous loss of *ARID2* can contribute to the phenotype of these cases. In agreement with this, reported *ARID2* variants argue for a loss-of-function mechanism (Shang et al., 2015;Bramswig et al., 2017;Van Paemel et al., 2017). This cluster therefore nicely illustrates that re-interpretation of old data and new findings can be helpful to gain insights in disease causing genetic mechanisms.

*Cluster 3 - 12q13.3q23.1*

11 individuals presenting with global developmental delay/intellectual disability, feeding difficulties and recurrent vomiting (4 males and 7 females) with overlapping deletions of chromosome bands 12q13.3q23.1 have been reported (**Supplementary Figure 2, Supplementary Table 5**)(Meinecke and Meinecke, 1987;Watson et al., 1989;Tocyap et al., 2006;Schluth et al., 2008;Yamanishi et al., 2008;Vergult et al., 2011;Lopez et al., 2012;Alesi et al., 2017;Alesi et al., 2019;Uehara et al., 2019). All parents were healthy, non-consanguineous and with normal karyotypes. Most of the pregnancies were uneventful but two showed IUGR (Schluth et al., 2008;Yamanishi et al., 2008). Nasal speech is commonly encountered feature of this deletion found in 8/8 affected individuals with the remaining individuals being pre-lingual infants or toddlers. Mild dysmorphic features were exhibited by the majority of the individuals with low set or rotated ears (9/9), broad, prominent or high forehead (6/6), short, broad, high or upturned nose (6/6), finger/hand deformities (7/11) and toe/feet deformities (8/11) (**Supplementary Table 5**).

Most reported individuals share a 1.62 Mb SRO at 12q15 which initially was reported to consist of the 5 genes *CNOT2*, *KCNMB4*, *PTPRR*, *TSPAN8* and *LGR5* (Vergult et al., 2011;Alesi et al., 2017), but which was recently further fined-mapped by two novel cases with deletions only harboring *CNOT2* but not the other candidate genes (Alesi et al., 2019;Uehara et al., 2019)*.* This gene is therefore likely involved in the pathogenesis of the 12q15 deletion syndrome (**Supplementary Table 3)**.

*CNOT2* encodes a subunit of the multi-component CCR4-NOT complex that is a master regulator of eukaryotic gene expression (Collart, 2016). Disruption of the CCR4-NOT complex results in dysregulation of global gene expression, and is associated with various human disease processes (Neely et al., 2010;Morita et al., 2011;Watanabe et al., 2014;Takahashi et al., 2015;Li et al., 2017). *CNOT2* is important for controlling cell viability through the maintenance of the structural integrity and enzymatic activity of the CCR4-NOT complex (Ito et al., 2011). Given its biological function haploinsufficiency of *CNOT2* might be causal for the neurological phenotypes of the 12q15 microdeletion syndrome. Interestingly, so far no individuals with pathogenic single nucleotide variants have been reported, and data from the gnomAD database show that the gene is highly intolerant to the loss of function on the basis of the number of protein truncating variants, with a pLI score of 1 (Karczewski et al., 2020). Therefore it seems likely that it will be only a matter of time till this gene will also become implicated in a monogenic disorder.

Given that a large number of affected individuals in this cluster also harbor deletions of *KCNMB4*, *PTPRR*, *TSPAN8* and *LGR5,* it is tempting to speculate that at least some the phenotypes could be influenced by haploinsufficiency of these genes. In 9 out of 11 individuals, *KCNMB4* is deleted. This gene encodes a neuronal MaxiK channel β4 subunit, which is highly expressed in brain, playing a role in the control of smooth muscle tone and neuronal excitability (Behrens et al., 2000;Alesi et al., 2017). It is proposed that neuronal β4 subunit with its pore forming α subunit is the molecular basis for toxin-insensitive MaxiK channels in the brain (Meera et al., 2000).

*PTPRR* encodes a member of the protein tyrosine phosphatase (PTP) family that participate in modulating differentiation, proliferation, and functioning of neurons (Schmitt et al., 2009). Chirivi et. al (Chirivi et al., 2007) indicated that *PTPRR* plays an essential role in maintaining mitogen-activated protein kinase (MAPK) activity in cerebellar Purkinje cells. The *PTPRR* proteins are physiological regulators of MAPK, and emerging evidence implicated MAPK signaling to be involved in cerebellar functions and cerebellar disorders (Hendriks et al., 2009;Erkens et al., 2015). A *PTPRR* deletion occurred in 8 of the described individuals, but none of them showed signs of ataxia.

In 8 of the 11 described individuals, *TSPAN8* is deleted. The protein encoded by this gene is a member of the transmembrane 4 superfamily, also known as the tetraspanin family. These proteins are reported to mediate signal transduction events, playing a role in the regulation of cell development, activation, growth and motility. *TSPAN8* has been implicated in schizophrenia and bipolar disorders (Scholz et al., 2010), tumor progression and metastasis (Kim et al., 2015) and neuronal function or development (Schartner et al., 2017).

Finally, *LGR5* was deleted in 8 individuals in this cluster. The protein encoded by *LGR5* gene is a leucine-rich repeat-containing receptor (LGR) and member of the G protein-coupled superfamily. The protein is involved in the Wnt signaling pathway and has been identified as a stem cell marker of the intestinal epithelium and the hair follicle (Barker et al., 2007). There is also strong genetic evidence that Wnt signaling plays critical roles in the regulation of epithelial stem cells in the intestinal tract (Haegebarth and Clevers, 2009). Mice deficient for *LGR5* exhibit malformation of tongue and lower jaws, causing newborns to swallow air, leading to early neonatal death (Morita et al., 2004). It would be interesting to investigate whether there is a correlation between feeding difficulties in 12q15 patients and intestinal dysfunction due to *LGR5* haploinsufficiency.

*Cluster 4 - 12q21.1q23.2*

Ten individuals (5 females, 5 males) have been reported with overlapping deletions involving 12q21.1-q23.2 (**Supplementary Figure 3, Supplementary Table 6**). In these individuals two SROs were identified, of 10.9 Mb at 12q21.2q21.33 and of 1.04 Mb at 12q21.33, that were shared between 9 and 6 individuals, respectively.

All reported individuals were born to non-consanguineous, healthy parents, by uncomplicated vaginal deliveries, except one assisted induction (Rauen et al., 2002), one vacuum extraction (Klein et al., 2005), and two c-sections (Matsumoto et al., 2014;McKenna et al., 2019). Family history was negative for developmental delay. Pregnancies were uneventful and with an average birth weight of 2.6 kg. Fetal ultrasound examinations revealed renal anomalies in four individuals (**Supplementary Table 3**).

Shared features observed in the affected individuals included global developmental delay/intellectual disability (100%), delayed speech (100%), and various dysmorphic features. Two of the most notable facial dysmorphic features were low set ears (100%) and a short upturned nose (75%). Furthermore, ectodermal abnormalities such as hyperkeratosis (85.71%) and thin hair (87.5%) were often described. Minor limb abnormalities of hand and feet were seen in 71.43% and 85.71% of the individuals respectively, the majority of which were transversal palmar creases and cutaneous 2-3 feet syndactyly. Cardiac, ocular and kidney abnormalities were also observed in 44.44%, 55.56% and 57.14% of the affected individuals, respectively (**Supplementary Table 6**).

Of the affected individuals, several were initially suspected to have a variety of other monogenic disorders. Brady et al. suspected Noonan syndrome (NS) in their case (Brady et al., 1999). Rauen et al. reported two individuals both presenting phenotypes reminiscent of cardio-facio-cutaneous (CFC) syndrome, consequently proposing a candidate region for this syndrome (Rauen et al., 2000;Rauen et al., 2002). James et al. reported an individual with a deletion of this candidate region, however this individual did not have many of the typical CFC symptoms. CFC and NS belong to a group of syndromes known as the RASopathies that are caused by germline mutation in genes encoding components of the Ras/mitogen-activated protein kinase (MAPK) pathway (James et al., 2005;Cao et al., 2017). None of the known related NS or CFC genes are however localized to 12q (Oliveira et al., 2015;Cano et al., 2016). Furthermore Cano et al. reported an individual with having an Oculo-Dento-Digital-Dysplasia (ODDD) like phenotype, while also noting the overlapping clinical presentation with CFC. ODDD is known to be linked to *GJA1* on chromosome 6q (Cano et al., 2016).

Klein et al. proposed a 12q specific phenotype when comparing their case with the two previously reported cases by Rauen et al (Rauen et al., 2000;Rauen et al., 2002;Klein et al., 2005). The phenotype consisted of global developmental delay/intellectual disability, facial dysmorphology (including a prominent forehead and short upturned nose), ectodermal abnormalities (such as sparse hair and hyperkeratosis pilaris/ulerythema ophryogenes), cardiac and renal malformations (Klein et al., 2005). They further suggested that, given its rarity and its distinct presence in all three individuals, the 12q21q23 region could be a candidate locus for hyperkeratosis pilaris/ulerythema ophryogenes.

In contrast to the other deletion clusters along 12q, the likely causative genes for the 12q21.1q23.2 regions are relatively poorly defined. The two SROs harbor a total of 29 genes (**Supplementary Table 3)**. A number of these genes, including *MYF6, LIN7A, KITLG, DCN* and *BTG1*, have been frequently discussed as possible contributors to the phenotypes of these patients, but it cannot be excluded that haploinsufficiency of other genes in this region might also be causative for the overlapping phenotypes. We here discuss some of the previously associated genes, and in addition a number of other genes that could additionally be involved.

*MYF6*, located at 12q21.31, encodes a DNA binding protein involved in muscle differentiation/determination and found to be related to Centronuclear Myopathy (CNM) (Kerst et al., 2000;Oliveira et al., 2015). Low muscle tone has only been reported in two of the ten individuals with deletions of this region (Brady et al., 1999;Oliveira et al., 2015) and hence the link between 12q21q23 deletion and myopathy is not very strong.

*LIN7A*, located at 12q21.31, encodes a scaffold protein playing a role in the CASK pathway that is important for synaptic function (Matsumoto et al., 2014). Matsumoto et al. described that *Lin7a* deficient mice have a delayed axonal growth and an abnormal neuronal migration, proposing that a mutation of this gene could impair cerebral development. Possibly this gene is connected to global developmental delay/intellectual disability observed in the majority of individuals.

*KITLG*, located at 12q21.32, encodes a protein involved in hematopoiesis, melanogenesis and gametogenesis. Mutations in this gene following an autosomal dominant inheritance pattern have been described to cause familial progressive hyper- and hypopigmentation, suggesting this gene as an important modulator of skin pigmentation (Amyere et al., 2011), and it has been suggested that KITLG may play a role in the ectodermic anomalies observed among individuals in this cluster (Klein et al., 2005;Oliveira et al., 2015). Furthermore mutations have been shown to lead to autosomal dominant, non-syndromic unilateral and asymmetric hearing loss, suggesting that *KITLG* also plays a role in hearing (Zazo Seco et al., 2015).

*DCN*, located at 12q21.33, was proposed by Al Maawali et al. to play a role in the ocular abnormalities observed in some of the affected individuals. *DCN* encodes the proteoglycan decorin which plays a role in collagen fibril assembly and is found in many connective tissues. Frame shift mutations of *DCN* have been reported in individuals with autosomal dominant congenital stromal corneal dystrophy. Furthermore a recent *Dcn* knockout mouse model showed that loss of *DCN* delayed the transition of hair cells to the active hair growing stage (Zhou et al., 2018), possibly reflecting the sparse/thin hair observed in many of the individuals.

*BTG1*, a gene located at 12q21.33, belongs to a family of anti-proliferative (APRO) genes that play an important role in the regulation of cell growth and cell differentiation in various cells such as T lymphocytes, fibroblasts, epithelial cells, and neuronal cells. Al-Maawali et al. 2014, proposed this as a critical gene for the development of the distinctive keratosis pilaris, as *BTG1* plays a role in proliferation control which is disturbed in keratosis pilaris.

*SYT1*, located at 12q21.2, is part of a family integral synaptic vesicle proteins which are required for the synchronous, calcium-dependent, neurotransmitter release. Furthermore it plays a role in the endocytosis of synaptic vesicles. Presumed dominant-acting, *de novo* missense variants in this gene have been reported in patients presenting with neurodevelopmental delay, hypotonia, movement disorders, motor stereotypies and congenital ophthalmic abnormalities (Baker et al., 2018).

*PPP1R12A*, located at 12q21.1-q21, is a developmental gene involved in cell adhesion, migration and morphogenesis. *De novo* loss-of-function variants were found in patients with mutations in this gene were reported having midline brain malformations, urogenital abnormalities or both (Hughes et al., 2020), suggesting that this gene is involved in a congenital malformation syndrome effecting the embryogenesis of the brain and genitourinary systems.

*PTPRQ*, located at 12q21.31, belongs to the type III receptor-like protein-tyrosine phosphatase family. These are involved in the regulation of survival and proliferation of cells as well as their subcellular architecture. Mutations in this gene have already been described as playing a role in recessive non-syndromic hearing loss. More recently mutations have also been described in the development of autosomal dominant non-syndromic hearing loss (Oziębło et al., 2019).

*PPFIA2*, 12q21.31, belongs to the liprin-alpha gene family. Liprins interact with members of the LAR family of transmembrane protein tyrosine phosphatases, these are important for axon guidance and mammary gland development (Serra-Pagès et al., 1998). A recent study examining a cohort of patients with unexplained intellectual disability proposed this gene as a novel candidate gene for intellectual disability (Uehara et al., 2016).

*TMTC2*, located at 12q21.31, encodes for an endoplasmic reticulum membrane protein involved in the cellular homeostasis of calcium. A family dyad with sensorineural hearing loss and auditory neuropathy spectrum disorder correlating this to a mutation of the TMTC2 gene has been reported (Guillen-Ahlers et al., 2018).

*DUSP6*, located at 12q21.33, encodes a protein belonging to a dual specificity protein phosphatase subfamily. These proteins negatively regulate members of the MAPK which is associated with cellular differentiation and proliferation. Mutations in this gene have been associated with congenital hypogonadotropic hypogonadism and anosmia (Miraoui et al., 2013).

Taken this all together, the phenotype causing genes for this cluster still need to be identified. Surprisingly, despite that *KITLG, PTPRQ* and *TMTC2* have been linked to hearing loss, hearing loss has only been reported for one individual with deletions of these regions. As several genes, including *PPFIA2*, *PPP1R12A, SYT1* and *LIN7A*, seem plausible candidates to explain the neurodevelopmental features in individuals with deletions in this cluster, characterization of additional cases with other deletions in this region of chromosome 12q will be crucial to further delineate the disease causing gene in this region.

Cluster 5 - 12q24.31q24.33

Twelve individuals (9 males, 3 females) have been reported with distal overlapping deletions at chromosome 12q24.31q24.33 (Sathya et al., 1999;van Karnebeek et al., 2002;Petek et al., 2003;Plotner et al., 2003;Niyazov et al., 2007;Baple et al., 2010;Al-Zahrani et al., 2011;Chouery et al., 2013;Kehrer et al., 2013;Qiao et al., 2013;Palumbo et al., 2015;Verhoeven et al., 2015;Labonne et al., 2016), with three SROs of 0.36 Mb at band 12q24.31, and of 1.6 Mb and 0.66 Mb at band 12q24.33, respectively. A single reported individual (Sathya et al., 1999) has a deletion that overlaps all three SROs (**Supplementary Figure 4**). Affected individuals were born to healthy, non-consanguineous parents in 7 cases. In one case, the mother was reported to have smoked throughout pregnancy (Niyazov et al., 2007), in two cases parents were consanguineous (Al-Zahrani et al., 2011;Kehrer et al., 2013), in one case the deletion was inherited from the father (Chouery et al., 2013) and one individual had familial dominant cystic kidney disease inherited from its father (Verhoeven et al., 2015). Pregnancies were uneventful in most cases. Prenatal ultrasound investigations revealed polyhydramnions and an enlarged fourth ventricle in one case (Sathya et al., 1999), and in another case polycystic and ectopic kidneys (Niyazov et al., 2007).

Global developmental delay/intellectual disability, behavioral issues (ranging from autism to hyperactivity and anxiety) as well as various dysmorphic features and genital abnormalities were often associated with 12q24.31-12q24.33 proximal and distal deletions (**Supplementary** **Table 7.1**, **Supplementary** **Table 7.2 and Supplementary Table 7.3**). All individuals with a 12q24.31 deletion were reported to have global developmental delay/intellectual disability and there were more reports of dysmorphic features compared to 12q24.33. Furthermore it is interesting to note that 80%, 50% and 25% of individuals with a 12q24.31 deletion had hypotonia, epilepsy or diabetes/hyperinsulinism, respectively, while none of the individuals with the 12q24.33 deletion had these symptoms. Obesity was reported in half of all individuals in both groups.

The 0.36 Mb SRO at 12q24.31 contains the genes *KDM2B*, *ORAI1*, *MORN3*, *TMEM120B*, *ROHOF*, and *SETD1B.*

Palumbo et al. and Labonne et al. both suggested a 12q24.3 microdeletion syndrome resulting in developmental delay, intellectual disabilities, seizures, behavioral problems and various dysmorphic anomalies. Both studies named *SETD1B* and *KDM2B* as possible causative genes, given their high haploinsufficiency scores and expression in various regions of the human brain.

*KDM2B* functions as a histone demethylase and has an essential role in neural development. *KDM2B* deficiency causes increased cell proliferation and cell death in neural progenitor cells (Fukuda et al., 2011). Recently, a missense variant in exon 15 of *KDM2B* was found to segregate in a Japanese family with a dominant inheritance of symptoms including Marfan syndrome-like minor physical anomalies, intellectual disability, and schizophrenia, possibly indicating that *KDM2B* might cause this phenotype (Yokotsuka-Ishida et al., 2021).

*SETD1B* is a component of histone methyl-transferase complex that contributes to the epigenetic control of chromatin structure and gene expression. Epigenetic mechanisms are increasingly linked to human disorders and in particular to genetic syndromes presenting typical clinical features such as neurodevelopmental delays and dysmorphic facial features. In recent studies, individuals with *de novo* *SETD1B* variants were described, presenting with epilepsy, developmental delay, intellectual disability and autism (Hiraide et al., 2018;Den et al., 2019;Hiraide et al., 2019;Krzyzewska et al., 2019;Roston et al., 2020). Recently, 36 cases with variants in *SETD1B* have been described and functionally investigated, further delineating the clinical and molecular phenotype of this newly recognized disorder (Weerts et al., 2021), pointing to a loss-of-function mechanism. The core clinical phenotype in this large cohort consisted of global developmental delay, language delay including regression, intellectual disability, autism and other behavioral issues, and variable epilepsy phenotypes. Also obesity was frequently encountered. Developmental delay preceded seizure onset, suggesting that *SETD1B* dysfunction impacts physiological neurodevelopment even in the absence of epileptic activity. Interestingly, males were significantly overrepresented and more severely affected, indicating that sex-linked traits could affect susceptibility to penetrance and the clinical spectrum of *SETD1B* variants.

From the other genes in the SRO, only missense variants in *ORAI1* have been linked to a phenotype of tubular aggregate myopathy (Nesin et al., 2014), but given the presumed gain-of-function mechanism, it seems unlikely that loss-of-function of this gene in the context of a deletion will contributed to a phenotype. It thus seems likely that the *SETD1B* aberration is the core defect in 12q24.31 deletion syndrome. Given the established role of *SETD1B* in epilepsy, this might explain why epilepsy is not found in individuals with more telomeric 12q24.33 deletions.

As only 4 deletions are reported that solely affect the more telomeric band 12q24.33, the candidate disease causing genes of this region are more poorly defined. The proximal 1.6 Mb SRO contains the genes *RIMBP2*, *STX2*, *RAN*, *ADGRD1*, *SFSWAP*, *MMP17*, *PUS1*, *ULK1*, and *EP400*, whereas the distal 0.66 Mb regions is more gene dense, containing the genes *FBRSL1*, *P2RX2*, *POLE*, *PXMP2*, *PGAM5*, *ANKLE2*, *GOLGA3*, *CHFR*, *ZNF605*, *ZNF26*, *ZNF84*, *ZNF140*, *ZNF891*, and *ZNF10* (**Supplementary Table 3**). Not much is known about the genes in both SROs.

A homozygous deletion of *STX2* has been associated in a single case with non-obstructive azoospermia (Nakamura et al., 2018). Homozygous variants in *PUS1* have been linked to myopathy, lactic acidosis, and sideroblastic anemia (MLASA), a rare autosomal recessive oxidative phosphorylation disorder specific to skeletal muscle and bone marrow (Bykhovskaya et al., 2004). *ULK1*, located at 12q24.33, is a protein kinase required for autophagy (Lin et al., 2012). *ULK1* is the ortholog of murine *Unc51.1*, a gene that has been shown to be one of the earliest genes involved in neuronal differentiation and is required for axonal elongation and granule cell axon formation via the neuronal endocytic pathway (Niyazov et al., 2007). It is tempting to speculate that deletion of this gene might therefore be implicated in the neurodevelopmental phenotypes of individuals with deletions of this region. Interestingly, *EP400* is a member of the NuA4 histone acetyltransferase (HAT) complex, and its knockdown in embryonic stem cells resulted in misregulation developmental genes (Fazzio et al., 2008). As many chromatin modifying enzymes result in neurodevelopmental phenotypes when mutant, this might be an interesting candidate gene to further explore in future studies.

Recently, truncating *de novo* variants in specific exons of *FBRSL1* have been found in three unrelated children with an overlapping syndromic phenotype with respiratory insufficiency, postnatal growth restriction, microcephaly, global developmental delay and other malformations (Ufartes et al., 2020). The function of *FBRSL1* is largely unknown.

*P2RX2*, located at 12q24.33, belongs to the family of purinoceptors for ATP and functions as a ligand-gated ion channel that binds to ATP and mediates synaptic transmission between neurons and from neurons to smooth muscle. ATP-gated ion channels, assembled from *P2RX2* receptor subunits, are believed to mediate adaptation to elevated sound levels (Housley et al., 2013). In agreement, heterozygous loss-of-function variants have been reported in autosomal dominant deafness (DFNA41) (Yan et al., 2013).

*POLE* encodes the catalytic subunit of DNA polymerase epsilon, of which heterozygous missense mutations cause increased susceptibility to colorectal cancer (Palles et al., 2013).

*ANKLE2* has a critical role in reassembly of the nuclear envelope at the onset of anaphase, and reduced ANKLE2 levels disrupts endoplasmic reticulum and nuclear envelope morphology. Bi-allelic variants in this gene have been linked to microcephaly (Link et al., 2019), but as heterozygous parents are healthy, it seems unlikely that heterozygosity of *ANKLE2* leads to a phenotype in 12q24 deletions.

Taken together, from the 23 genes annotated in the telomeric 12q24.33 deletions, only for *ULK1*, *EP400*, *FBRSL1* and *P2RX2* a plausible role in the pathogenesis could be hypothesized. Study of additional deletions in this region might help to further delineate the associated phenotypes.

Interestingly, Qiao et al. described a deletion case at 12q24.31, which does not overlap any of the three SROs discussed in this section. This deletion contains the genes *GPR81, GPR109A* and *GRP109B*, which are G-protein couple receptors (also referred to as HCA receptors) that help maintain homeostasis under changing metabolic and dietary conditions, by controlling metabolic, immune, and other body functions. In a *Gpr109a* knockout mouse model, hepatic steatosis and age-related obesity was observed (Jadeja et al., 2019). Whether deletions of these genes contributes to the phenotype remains to be determined.

**References**

Adam, M.P., Mehta, A., Knight, L., Hall, D.E., and Rossi, M.R. (2010). A family with a 1.17 Mb deletion of 12q12: refining genotype-phenotype correlation. *Am J Med Genet A* 152a**,** 2394-2398.

Aihara, K., Kuroda, S., Kanayama, N., Matsuyama, S., Tanizawa, K., and Horie, M. (2003). A neuron-specific EGF family protein, NELL2, promotes survival of neurons through mitogen-activated protein kinases. *Brain Res Mol Brain Res* 116**,** 86-93.

Al-Zahrani, J., Al-Dosari, N., Abudheim, N., Alshidi, T.A., Colak, D., Al-Habit, O., et al. (2011). Chromosome 12q24.31-q24.33 deletion causes multiple dysmorphic features and developmental delay: First mosaic patient and overview of the phenotype related to 12q24qter defects. *Molecular Cytogenetics* 4**,** 9-9.

Alesi, V., Loddo, S., Calì, F., Orlando, V., Genovese, S., Ferretti, D., et al. (2019). A heterozygous, intragenic deletion of CNOT2 recapitulates the phenotype of 12q15 deletion syndrome. *Am J Med Genet A* 179**,** 1615-1621.

Alesi, V., Loddo, S., Grispo, M., Riccio, S., Montella, A.C., Dallapiccola, B., et al. (2017). Reassessment of the 12q15 deletion syndrome critical region. *Eur J Med Genet* 60**,** 220-223.

Amyere, M., Vogt, T., Hoo, J., Brandrup, F., Bygum, A., Boon, L., et al. (2011). KITLG mutations cause familial progressive hyper- and hypopigmentation. *J Invest Dermatol* 131**,** 1234-1239.

Baker, K., Gordon, S.L., Melland, H., Bumbak, F., Scott, D.J., Jiang, T.J., et al. (2018). SYT1-associated neurodevelopmental disorder: a case series. *Brain* 141**,** 2576-2591.

Baple, E., Palmer, R., and Hennekam, R.C.M. (2010). A Microdeletion at 12q24.31 Can Mimic Beckwith-Wiedemann Syndrome Neonatally. *Molecular Syndromology* 1**,** 42-45.

Barker, N., Van Es, J.H., Kuipers, J., Kujala, P., Van Den Born, M., Cozijnsen, M., et al. (2007). - Identification of stem cells in small intestine and colon by marker gene Lgr5. - 449**,** - 1007.

Bassuk, A.G., and Sherr, E.H. (2015). A de novo mutation in PRICKLE1 in fetal agenesis of the corpus callosum and polymicrogyria. *Journal of neurogenetics* 29**,** 174-177.

Basu, A., Wilkinson, F.H., Colavita, K., Fennelly, C., and Atchison, M.L. (2014). YY1 DNA binding and interaction with YAF2 is essential for Polycomb recruitment. *Nucleic Acids Res* 42**,** 2208-2223.

Behrens, R., Nolting, A., Reimann, F., Schwarz, M., Waldschutz, R., and Pongs, O. (2000). hKCNMB3 and hKCNMB4, cloning and characterization of two members of the large-conductance calcium-activated potassium channel beta subunit family. *FEBS Lett* 474**,** 99-106.

Brady, A., Elsawi, M., Jamieson, C., Marks, K., Jeffery, S., Patton, M., et al. (1999). Clinical and molecular findings in a patient with a deletion on the long arm of chromosome 12. *Journal of Medical Genetics* 36**,** 939-941.

Bramswig, N.C., Caluseriu, O., Lüdecke, H.J., Bolduc, F.V., Noel, N.C., Wieland, T., et al. (2017). Heterozygosity for ARID2 loss-of-function mutations in individuals with a Coffin-Siris syndrome-like phenotype. *Hum Genet* 136**,** 297-305.

Bykhovskaya, Y., Casas, K., Mengesha, E., Inbal, A., and Fischel-Ghodsian, N. (2004). Missense mutation in pseudouridine synthase 1 (PUS1) causes mitochondrial myopathy and sideroblastic anemia (MLASA). *Am J Hum Genet* 74**,** 1303-1308.

Cano, M., Trapasso, J., Trapasso, T., and Matalon, R. (2016). 12 q deletion with oculodentodigital dysplasia -like phenotype. *Clinical Case Reports and Reviews* 2.

Cao, H., Alrejaye, N., Klein, O.D., Goodwin, A.F., and Oberoi, S. (2017). A review of craniofacial and dental findings of the RASopathies. *Orthod Craniofac Res* 20 Suppl 1**,** 32-38.

Carlsen, E., Frengen, E., Fannemel, M., and Misceo, D. (2015). Haploinsufficiency of ANO6, NELL2 and DBX2 in a boy with intellectual disability and growth delay. *Am J Med Genet A* 167a**,** 1890-1896.

Chirivi, R.G., Noordman, Y.E., Van Der Zee, C.E., and Hendriks, W.J. (2007). Altered MAP kinase phosphorylation and impaired motor coordination in PTPRR deficient mice. *J Neurochem* 101**,** 829-840.

Chouery, E., Choucair, N., Abou Ghoch, J., El Sabbagh, S., Corbani, S., and Mégarbané, A. (2013). Report on a Patient with a 12q24.31 Microdeletion Inherited from an Insulin-Dependent Diabetes Mellitus Father. *Molecular Syndromology* 4**,** 136-142.

Collart, M.A. (2016). The Ccr4-Not complex is a key regulator of eukaryotic gene expression. *Wiley Interdiscip Rev RNA* 7**,** 438-454.

Den, K., Kato, M., Yamaguchi, T., Miyatake, S., Takata, A., Mizuguchi, T., et al. (2019). A novel de novo frameshift variant in SETD1B causes epilepsy. *J Hum Genet* 64**,** 821-827.

Erkens, M., Tanaka-Yamamoto, K., Cheron, G., Márquez-Ruiz, J., Prigogine, C., Schepens, J.T., et al. (2015). Protein tyrosine phosphatase receptor type R is required for Purkinje cell responsiveness in cerebellar long-term depression. *Mol Brain* 8**,** 1.

Failla, P., Romano, C., Reitano, S., Di Benedetto, D., Grillo, L., Fichera, M., et al. (2008). 12q12 deletion: a new patient contributing to genotype-phenotype correlation. *Am J Med Genet A* 146a**,** 1354-1357.

Fazzio, T.G., Huff, J.T., and Panning, B. (2008). An RNAi screen of chromatin proteins identifies Tip60-p400 as a regulator of embryonic stem cell identity. *Cell* 134**,** 162-174.

Fukuda, T., Tokunaga, A., Sakamoto, R., and Yoshida, N. (2011). Fbxl10/Kdm2b deficiency accelerates neural progenitor cell death and leads to exencephaly. *Mol Cell Neurosci* 46**,** 614-624.

Gabriele, M., Vulto-Van Silfhout, A.T., Germain, P.L., Vitriolo, A., Kumar, R., Douglas, E., et al. (2017). YY1 Haploinsufficiency Causes an Intellectual Disability Syndrome Featuring Transcriptional and Chromatin Dysfunction. *Am J Hum Genet* 100**,** 907-925.

Gibbs, B.C., Damerla, R.R., Vladar, E.K., Chatterjee, B., Wan, Y., Liu, X., et al. (2016). Prickle1 mutation causes planar cell polarity and directional cell migration defects associated with cardiac outflow tract anomalies and other structural birth defects. *Biol Open* 5**,** 323-335.

Guillen-Ahlers, H., Erbe, C.B., Chevalier, F.D., Montoya, M.J., Zimmerman, K.D., Langefeld, C.D., et al. (2018). TMTC2 variant associated with sensorineural hearing loss and auditory neuropathy spectrum disorder in a family dyad. *Mol Genet Genomic Med* 6**,** 653-659.

Haegebarth, A., and Clevers, H. (2009). Wnt Signaling, Lgr5, and Stem Cells in the Intestine and Skin. *The American Journal of Pathology* 174**,** 715-721.

Hendriks, W.J., Dilaver, G., Noordman, Y.E., Kremer, B., and Fransen, J.A. (2009). PTPRR protein tyrosine phosphatase isoforms and locomotion of vesicles and mice. *Cerebellum* 8**,** 80-88.

Hiraide, T., Hattori, A., Ieda, D., Hori, I., Saitoh, S., Nakashima, M., et al. (2019). - De novo variants in SETD1B cause intellectual disability, autism spectrum disorder, and epilepsy with myoclonic absences. - 4**,** - 481.

Hiraide, T., Nakashima, M., Yamoto, K., Fukuda, T., Kato, M., Ikeda, H., et al. (2018). - De novo variants in SETD1B are associated with intellectual disability, epilepsy and autism. - 137**,** - 104.

Housley, G.D., Morton-Jones, R., Vlajkovic, S.M., Telang, R.S., Paramananthasivam, V., Tadros, S.F., et al. (2013). ATP-gated ion channels mediate adaptation to elevated sound levels. *Proc Natl Acad Sci U S A* 110**,** 7494-7499.

Hughes, J.J., Alkhunaizi, E., Kruszka, P., Pyle, L.C., Grange, D.K., Berger, S.I., et al. (2020). Loss-of-Function Variants in PPP1R12A: From Isolated Sex Reversal to Holoprosencephaly Spectrum and Urogenital Malformations. *Am J Hum Genet* 106**,** 121-128.

Ito, K., Inoue, T., Yokoyama, K., Morita, M., Suzuki, T., and Yamamoto, T. (2011). CNOT2 depletion disrupts and inhibits the CCR4-NOT deadenylase complex and induces apoptotic cell death. *Genes Cells* 16**,** 368-379.

Jadeja, R.N., Jones, M.A., Fromal, O., Powell, F.L., Khurana, S., Singh, N., et al. (2019). Loss of GPR109A/HCAR2 induces aging-associated hepatic steatosis. *Aging (Albany NY)* 11**,** 386-400.

James, P.A., Oei, P., Ng, D., Kannu, P., and Aftimos, S. (2005). Another case of interstitial del(12) involving the proposed cardio-facio-cutaneous candidate region. *Am J Med Genet A* 136**,** 12-16.

Jaworski, A., Tom, I., Tong, R.K., Gildea, H.K., Koch, A.W., Gonzalez, L.C., et al. (2015). Operational redundancy in axon guidance through the multifunctional receptor Robo3 and its ligand NELL2. *Science* 350**,** 961-965.

Karczewski, K.J., Francioli, L.C., Tiao, G., Cummings, B.B., Alföldi, J., Wang, Q., et al. (2020). The mutational constraint spectrum quantified from variation in 141,456 humans. *Nature* 581**,** 434-443.

Kazerounian, S., and Aho, S. (2003). Characterization of periphilin, a widespread, highly insoluble nuclear protein and potential constituent of the keratinocyte cornified envelope. *J Biol Chem* 278**,** 36707-36717.

Kehrer, M., Singer, S., Grasshoff, U., Schaferhoff, K., Bonin, M., Riess, O., et al. (2013). 12q24.33 deletion: report of a patient with intellectual disability and review of the literature. *Am J Med Genet A* 161a**,** 1409-1413.

Kerst, B., Mennerich, D., Schuelke, M., Stoltenburg-Didinger, G., Von Moers, A., Gossrau, R., et al. (2000). Heterozygous myogenic factor 6 mutation associated with myopathy and severe course of Becker muscular dystrophy. *Neuromuscul Disord* 10**,** 572-577.

Kim, H., Ha, C.M., Choi, J., Choi, E.J., Jeon, J., Kim, C., et al. (2002). Ontogeny and the possible function of a novel epidermal growth factor-like repeat domain-containing protein, NELL2, in the rat brain. *J Neurochem* 83**,** 1389-1400.

Kim, T.K., Park, C.S., Jeoung, M.H., Lee, W.R., Go, N.K., Choi, J.R., et al. (2015). Generation of a human antibody that inhibits TSPAN8-mediated invasion of metastatic colorectal cancer cells. *Biochem Biophys Res Commun* 468**,** 774-780.

Klein, O.D., Cotter, P.D., Schmidt, A.M., Bick, D.P., Tidyman, W.E., Albertson, D.G., et al. (2005). Interstitial deletion of chromosome 12q: genotype-phenotype correlation of two patients utilizing array comparative genomic hybridization. *Am J Med Genet A* 138**,** 349-354.

Krzyzewska, I.M., Maas, S.M., Henneman, P., Lip, K.V.D., Venema, A., Baranano, K., et al. (2019). - A genome-wide DNA methylation signature for SETD1B-related syndrome. - 11.

Labonne, J.D., Lee, K.H., Iwase, S., Kong, I.K., Diamond, M.P., Layman, L.C., et al. (2016). An atypical 12q24.31 microdeletion implicates six genes including a histone demethylase KDM2B and a histone methyltransferase SETD1B in syndromic intellectual disability. *Hum Genet* 135**,** 757-771.

Lacin, H., Zhu, Y., Wilson, B.A., and Skeath, J.B. (2009). dbx mediates neuronal specification and differentiation through cross-repressive, lineage-specific interactions with eve and hb9. *Development* 136**,** 3257-3266.

Lanuza, G.M., Gosgnach, S., Pierani, A., Jessell, T.M., and Goulding, M. (2004). Genetic identification of spinal interneurons that coordinate left-right locomotor activity necessary for walking movements. *Neuron* 42**,** 375-386.

Li, X., Morita, M., Kikuguchi, C., Takahashi, A., Suzuki, T., and Yamamoto, T. (2017). Adipocyte-specific disruption of mouse Cnot3 causes lipodystrophy. *FEBS Lett* 591**,** 358-368.

Lin, S.Y., Li, T.Y., Liu, Q., Zhang, C., Li, X., Chen, Y., et al. (2012). GSK3-TIP60-ULK1 signaling pathway links growth factor deprivation to autophagy. *Science* 336**,** 477-481.

Link, N., Chung, H., Jolly, A., Withers, M., Tepe, B., Arenkiel, B.R., et al. (2019). Mutations in ANKLE2, a ZIKA Virus Target, Disrupt an Asymmetric Cell Division Pathway in Drosophila Neuroblasts to Cause Microcephaly. *Dev Cell* 51**,** 713-729.e716.

Lopez, E., Callier, P., Cormier-Daire, V., Lacombe, D., Moncla, A., Bottani, A., et al. (2012). Search for a gene responsible for Floating-Harbor syndrome on chromosome 12q15q21.1. *Am J Med Genet A* 158A**,** 333-339.

Mastrangelo, M., Tolve, M., Martinelli, M., Di Noia, S.P., Parrini, E., and Leuzzi, V. (2018). PRICKLE1-related early onset epileptic encephalopathy. *Am J Med Genet A* 176**,** 2841-2845.

Matsumoto, A., Mizuno, M., Hamada, N., Nozaki, Y., Jimbo, E.F., Momoi, M.Y., et al. (2014). LIN7A depletion disrupts cerebral cortex development, contributing to intellectual disability in 12q21-deletion syndrome. *PLoS One* 9**,** e92695.

Mckenna, C.S., Saxena, N., Dabir, T.A., Jones, J., Smith, G., and Morrison, P.J. (2019). Phenotypic delineation of a 12q21 deletion syndrome. *Clin Dysmorphol* 28**,** 198-201.

Meera, P., Wallner, M., and Toro, L. (2000). A neuronal beta subunit (KCNMB4) makes the large conductance, voltage- and Ca2+-activated K+ channel resistant to charybdotoxin and iberiotoxin. *Proc Natl Acad Sci U S A* 97**,** 5562-5567.

Meinecke, P., and Meinecke, R. (1987). Multiple malformation syndrome including cleft lip and palate and cardiac abnormalities due to an interstitial deletion of chromosome 12q. *J Med Genet* 24**,** 187.

Millington-Burgess, S.L., and Harper, M.T. (2019). Gene of the issue: ANO6 and Scott Syndrome. *Platelets***,** 1-4.

Miraoui, H., Dwyer, A.A., Sykiotis, G.P., Plummer, L., Chung, W., Feng, B., et al. (2013). Mutations in FGF17, IL17RD, DUSP6, SPRY4, and FLRT3 are identified in individuals with congenital hypogonadotropic hypogonadism. *Am J Hum Genet* 92**,** 725-743.

Miyake, N., Tonoki, H., Gallego, M., Harada, N., Shimokawa, O., Yoshiura, K., et al. (2004). Phenotype-genotype correlation in two patients with 12q proximal deletion. *J Hum Genet* 49**,** 282-284.

Morita, H., Mazerbourg, S., Bouley, D.M., Luo, C.W., Kawamura, K., Kuwabara, Y., et al. (2004). Neonatal lethality of LGR5 null mice is associated with ankyloglossia and gastrointestinal distension. *Mol Cell Biol* 24**,** 9736-9743.

Morita, M., Oike, Y., Nagashima, T., Kadomatsu, T., Tabata, M., Suzuki, T., et al. (2011). Obesity resistance and increased hepatic expression of catabolism-related mRNAs in Cnot3+/- mice. *Embo j* 30**,** 4678-4691.

Nakamura, S., Kobori, Y., Ueda, Y., Tanaka, Y., Ishikawa, H., Yoshida, A., et al. (2018). STX2 is a causative gene for nonobstructive azoospermia. *Hum Mutat* 39**,** 830-833.

Neely, G.G., Kuba, K., Cammarato, A., Isobe, K., Amann, S., Zhang, L., et al. (2010). A global in vivo Drosophila RNAi screen identifies NOT3 as a conserved regulator of heart function. *Cell* 141**,** 142-153.

Nesin, V., Wiley, G., Kousi, M., Ong, E.C., Lehmann, T., Nicholl, D.J., et al. (2014). Activating mutations in STIM1 and ORAI1 cause overlapping syndromes of tubular myopathy and congenital miosis. *Proc Natl Acad Sci U S A* 111**,** 4197-4202.

Niyazov, D.M., Nawaz, Z., Justice, A.N., Toriello, H.V., Martin, C.L., and Adam, M.P. (2007). Genotype/phenotype correlations in two patients with 12q subtelomere deletions. *Am J Med Genet A* 143a**,** 2700-2705.

Oliveira, R., Pereira, C., Melo, J.B., Mesquita, S., Venancio, M., Carreira, I.M., et al. (2015). 12q21.2q22 deletion: a new patient. *Am J Med Genet A* 167a**,** 1877-1883.

Oziębło, D., Sarosiak, A., Leja, M.L., Budde, B.S., Tacikowska, G., Di Donato, N., et al. (2019). First confirmatory study on PTPRQ as an autosomal dominant non-syndromic hearing loss gene. *J Transl Med* 17**,** 351.

Pak, J.S., Deloughery, Z.J., Wang, J., Acharya, N., Park, Y., Jaworski, A., et al. (2020). NELL2-Robo3 complex structure reveals mechanisms of receptor activation for axon guidance. *Nat Commun* 11**,** 1489.

Palles, C., Cazier, J.B., Howarth, K.M., Domingo, E., Jones, A.M., Broderick, P., et al. (2013). Germline mutations affecting the proofreading domains of POLE and POLD1 predispose to colorectal adenomas and carcinomas. *Nat Genet* 45**,** 136-144.

Palumbo, O., Palumbo, P., Delvecchio, M., Palladino, T., Stallone, R., Crisetti, M., et al. (2015). Microdeletion of 12q24.31: report of a girl with intellectual disability, stereotypies, seizures and facial dysmorphisms. *Am J Med Genet A* 167a**,** 438-444.

Petek, E., Windpassinger, C., Mach, M., Rauter, L., Scherer, S.W., Wagner, K., et al. (2003). Molecular characterization of a 12q22-q24 deletion associated with congenital deafness: confirmation and refinement of the DFNA25 locus. *Am J Med Genet A* 117a**,** 122-126.

Plotner, P.L., Smith, J.L., and Northrup, H. (2003). Deletion 12q: a second patient with 12q24.31q24.32 deletion. *Am J Med Genet A* 118a**,** 350-352.

Qiao, Y., Tyson, C., Hrynchak, M., Lopez-Rangel, E., Hildebrand, J., Martell, S., et al. (2013). Clinical application of 2.7M Cytogenetics array for CNV detection in subjects with idiopathic autism and/or intellectual disability. *Clin Genet* 83**,** 145-154.

Rapley, E.A., Hargrave, D., Persinguhe, N., Barfoot, R., Moore, I., Radford, M., et al. (2001). Case of interstitial 12q deletion in association with Wilms tumor. *Am J Med Genet* 104**,** 246-249.

Rauen, K.A., Albertson, D.G., Pinkel, D., and Cotter, P.D. (2002). Additional patient with del(12)(q21.2q22): further evidence for a candidate region for cardio-facio-cutaneous syndrome? *Am J Med Genet* 110**,** 51-56.

Rauen, K.A., Cotter, P.D., Bitts, S.M., Cox, V.A., and Golabi, M. (2000). Cardio-facio-cutaneous syndrome phenotype in an individual with an interstitial deletion of 12q: identification of a candidate region for CFC syndrome. *Am J Med Genet* 93**,** 219-222.

Roston, A., Evans, D., Gill, H., Mckinnon, M., Isidor, B., Cogne, B., et al. (2020). SETD1B-associated neurodevelopmental disorder. *J Med Genet*.

Sathya, P., Tomkins, D.J., Freeman, V., Paes, B., and Nowaczyk, M.J. (1999). De novo deletion 12q: report of a patient with 12q24.31q24.33 deletion. *Am J Med Genet* 84**,** 116-119.

Schartner, C., Scholz, C.J., Weber, H., Post, A., Freudenberg, F., Grunewald, L., et al. (2017). The regulation of tetraspanin 8 gene expression-A potential new mechanism in the pathogenesis of bipolar disorder. *Am J Med Genet B Neuropsychiatr Genet* 174**,** 740-750.

Schluth, C., Gesny, R., Borck, G., Redon, R., Abadie, V., Kleinfinger, P., et al. (2008). New case of interstitial deletion 12(q15-q21.2) in a girl with facial dysmorphism and mental retardation. *Am J Med Genet A* 146a**,** 93-96.

Schmitt, I., Bitoun, E., and Manto, M. (2009). PTPRR, cerebellum, and motor coordination. *Cerebellum* 8**,** 71-73.

Scholz, C.J., Jacob, C.P., Buttenschon, H.N., Kittel-Schneider, S., Boreatti-Hummer, A., Zimmer, M., et al. (2010). Functional variants of TSPAN8 are associated with bipolar disorder and schizophrenia. *Am J Med Genet B Neuropsychiatr Genet* 153b**,** 967-972.

Serra-Pagès, C., Medley, Q.G., Tang, M., Hart, A., and Streuli, M. (1998). Liprins, a family of LAR transmembrane protein-tyrosine phosphatase-interacting proteins. *J Biol Chem* 273**,** 15611-15620.

Shang, L., Cho, M.T., Retterer, K., Folk, L., Humberson, J., Rohena, L., et al. (2015). Mutations in ARID2 are associated with intellectual disabilities. *neurogenetics* 16**,** 307-314.

Shimojo, M., and Hersh, L.B. (2003). REST/NRSF-interacting LIM domain protein, a putative nuclear translocation receptor. *Mol Cell Biol* 23**,** 9025-9031.

Shimojo, M., and Hersh, L.B. (2006). Characterization of the REST/NRSF-interacting LIM domain protein (RILP): localization and interaction with REST/NRSF. *J Neurochem* 96**,** 1130-1138.

Stanton, S.E., Mcreynolds, L.J., Evans, T., and Schreiber-Agus, N. (2006). Yaf2 inhibits caspase 8-mediated apoptosis and regulates cell survival during zebrafish embryogenesis. *J Biol Chem* 281**,** 28782-28793.

Takahashi, A., Adachi, S., Morita, M., Tokumasu, M., Natsume, T., Suzuki, T., et al. (2015). Post-transcriptional Stabilization of Ucp1 mRNA Protects Mice from Diet-Induced Obesity. *Cell Rep* 13**,** 2756-2767.

Tocyap, M.L., Azar, N., Chen, T., and Wiggs, J. (2006). Clinical and molecular characterization of a patient with an interstitial deletion of chromosome 12q15-q23 and peripheral corneal abnormalities. *Am J Ophthalmol* 141**,** 566-567.

Todd, B.P., and Bassuk, A.G. (2018). A de novo mutation in PRICKLE1 associated with myoclonic epilepsy and autism spectrum disorder. *J Neurogenet* 32**,** 313-315.

Tonoki, H., Saitoh, S., and Kobayashi, K. (1998). Patient with del(12)(q12q13.12) manifesting abnormalities compatible with Noonan syndrome. *Am J Med Genet* 75**,** 416-418.

Uehara, D.T., Hayashi, S., Okamoto, N., Mizuno, S., Chinen, Y., Kosaki, R., et al. (2016). SNP array screening of cryptic genomic imbalances in 450 Japanese subjects with intellectual disability and multiple congenital anomalies previously negative for large rearrangements. *J Hum Genet* 61**,** 335-343.

Uehara, T., Takenouchi, T., Yamaguchi, Y., Daimon, Y., Suzuki, H., Sakaguchi, Y., et al. (2019). CNOT2 as the critical gene for phenotypes of 12q15 microdeletion syndrome. *Am J Med Genet A* 179**,** 659-662.

Ufartes, R., Berger, H., Till, K., Salinas, G., Sturm, M., Altmüller, J., et al. (2020). De novo mutations in FBRSL1 cause a novel recognizable malformation and intellectual disability syndrome. *Hum Genet* 139**,** 1363-1379.

Van Karnebeek, C.D., Koevoets, C., Sluijter, S., Bijlsma, E.K., Smeets, D.F., Redeker, E.J., et al. (2002). Prospective screening for subtelomeric rearrangements in children with mental retardation of unknown aetiology: the Amsterdam experience. *J Med Genet* 39**,** 546-553.

Van Paemel, R., De Bruyne, P., Van Der Straaten, S., D'hondt, M., Fränkel, U., Dheedene, A., et al. (2017). Confirmation of an ARID2 defect in SWI/SNF-related intellectual disability. *Am J Med Genet A* 173**,** 3104-3108.

Vergult, S., Krgovic, D., Loeys, B., Lyonnet, S., Lieden, A., Anderlid, B.M., et al. (2011). Nasal speech and hypothyroidism are common hallmarks of 12q15 microdeletions. *Eur J Hum Genet* 19**,** 1032-1037.

Verheul, T.C.J., Van Hijfte, L., Perenthaler, E., and Barakat, T.S. (2020). The Why of YY1: Mechanisms of Transcriptional Regulation by Yin Yang 1. *Front Cell Dev Biol* 8**,** 592164.

Verhoeven, W.M., Egger, J.I., Van Den Bergh, J.P., Van Beek, R., Kleefstra, T., and De Leeuw, N. (2015). A 12q24.31 interstitial deletion in an adult male with MODY3: neuropsychiatric and neuropsychological characteristics. *Am J Med Genet A* 167a**,** 169-173.

Wang, H., Gao, M.X., Li, L., Wang, B., Hori, N., and Sato, K. (2007). Isolation, expression, and characterization of the human ZCRB1 gene mapped to 12q12. *Genomics* 89**,** 59-69.

Watanabe, C., Morita, M., Hayata, T., Nakamoto, T., Kikuguchi, C., Li, X., et al. (2014). Stability of mRNA influences osteoporotic bone mass via CNOT3. *Proc Natl Acad Sci U S A* 111**,** 2692-2697.

Watson, M.S., Mcallister-Barton, L., Mahoney, M.J., and Breg, W.R. (1989). Deletion (12)(q15q21.2). *J Med Genet* 26**,** 343-344.

Weerts, M.J.A., Lanko, K., Guzmán-Vega, F.J., Jackson, A., Ramakrishnan, R., Cardona-Londoño, K.J., et al. (2021). Delineating the molecular and phenotypic spectrum of the SETD1B-related syndrome. *Genet Med*.

Weng, Y., Luo, X., and Hou, L. (2018). Deletion at 12q12 increases the risk of developmental delay and intellectual disability. *Ann Hum Genet* 82**,** 482-487.

Won, H., De La Torre-Ubieta, L., Stein, J.L., Parikshak, N.N., Huang, J., Opland, C.K., et al. (2016). Chromosome conformation elucidates regulatory relationships in developing human brain. *Nature* 538**,** 523-527.

Yamanishi, T., Nishio, J., Miya, S., Okamoto, N., Takahashi, A., Toribe, Y., et al. (2008). 12q interstitial deletion with bilateral cleft lip and palate: case report and literature review. *Cleft Palate Craniofac J* 45**,** 325-328.

Yan, D., Zhu, Y., Walsh, T., Xie, D., Yuan, H., Sirmaci, A., et al. (2013). Mutation of the ATP-gated P2X(2) receptor leads to progressive hearing loss and increased susceptibility to noise. *Proc Natl Acad Sci U S A* 110**,** 2228-2233.

Yokotsuka-Ishida, S., Nakamura, M., Tomiyasu, Y., Nagai, M., Kato, Y., Tomiyasu, A., et al. (2021). Positional cloning and comprehensive mutation analysis identified a novel KDM2B mutation in a Japanese family with minor malformations, intellectual disability, and schizophrenia. *J Hum Genet*.

Zazo Seco, C., Serrão De Castro, L., Van Nierop, J.W., Morín, M., Jhangiani, S., Verver, E.J., et al. (2015). Allelic Mutations of KITLG, Encoding KIT Ligand, Cause Asymmetric and Unilateral Hearing Loss and Waardenburg Syndrome Type 2. *Am J Hum Genet* 97**,** 647-660.

Zhou, L., Jing, J., Wang, H., Wu, X., and Lu, Z. (2018). Decorin promotes proliferation and migration of ORS keratinocytes and maintains hair anagen in mice. *Exp Dermatol* 27**,** 1237-1244.
